# Preoperative Midazolam and Patient-Centered Outcomes of Older Patients: The I-PROMOTE Randomized Clinical Trial

**DOI:** 10.1001/jamasurg.2023.6479

**Published:** 2023-12-20

**Authors:** Ana Kowark, András P. Keszei, Gerhard Schneider, Stefanie Pilge, Frederick Schneider, David P. Obert, Marie-Therese Georgii, Markus Heim, Rolf Rossaint, Sebastian Ziemann, Julia van Waesberghe, Michael Czaplik, Friedrich K. Pühringer, Christian Minarski, Verena May, Tobias Malisi, Berthold Drexler, Carmen Maria Ring, Phillip Engler, Roman Tilly, Petra Bischoff, Ulrich Frey, Maria Wittmann, Martin Soehle, Thomas Saller, Peter Kienbaum, Moritz Kretzschmar, Mark Coburn

**Affiliations:** 1 Department of Anaesthesiology and Intensive Care Medicine, University Hospital Bonn, Bonn, Germany; 2 Department of Anesthesiology, Medical Faculty University Hospital RWTH Aachen, Aachen, Germany; 3 Center for Translational & Clinical Research Aachen, Medical Faculty RWTH Aachen University, Aachen, Germany; 4 Department of Anesthesiology and Intensive Care, Technical University of Munich, School of Medicine, Munich, Germany; 5 Department for Anaesthesiology, Intensive Care, Emergency Medicine, Pain Therapy and Palliative Care, Kreiskliniken Reutlingen, Reutlingen, Germany; 6 Department of Anaesthesiology and Intensive Care, University Hospital Tübingen, Tübingen, Germany; 7 Department of Radiology, University Hospital Tübingen, Tübingen, Germany; 8 Department of Anaesthesiology, Surgical Intensive Care, Pain and Palliative Care, Marien Hospital Herne, University Hospital of Ruhr University Bochum, Herne, Germany; 9 Department of Anaesthesiology, University Hospital, LMU Munich, Munich, Germany; 10 Department of Anaesthesiology, University Hospital Düsseldorf, Düsseldorf, Germany; 11 Department of Anaesthesiology and Intensive Care Medicine, Otto-von-Guericke-University Magdeburg, Magdeburg, Germany

## Abstract

**Importance:**

The effect of oral midazolam premedication on patient satisfaction in older patients undergoing surgery is unclear, despite its widespread use.

**Objective:**

To determine the differences in global perioperative satisfaction in patients with preoperative administration of oral midazolam compared with placebo.

**Design, Setting, and Participants:**

This double-blind, parallel-group, placebo-controlled randomized clinical trial was conducted in 9 German hospitals between October 2017 and May 2019 (last follow-up, June 24, 2019). Eligible patients aged 65 to 80 years who were scheduled for elective inpatient surgery for at least 30 minutes under general anesthesia and with planned extubation were enrolled. Data were analyzed from November 2019 to December 2020.

**Interventions:**

Patients were randomized to receive oral midazolam, 3.75 mg (n = 309), or placebo (n = 307) 30 to 45 minutes prior to anesthesia induction.

**Main Outcomes and Measures:**

The primary outcome was global patient satisfaction evaluated using the self-reported Evaluation du Vécu de l’Anesthésie Generale (EVAN-G) questionnaire on the first postoperative day. Key secondary outcomes included sensitivity and subgroup analyses of the primary outcome, perioperative patient vital data, adverse events, serious complications, and cognitive and functional recovery up to 30 days postoperatively.

**Results:**

Among 616 randomized patients, 607 were included in the primary analysis. Of these, 377 (62.1%) were male, and the mean (SD) age was 71.9 (4.4) years. The mean (SD) global index of patient satisfaction did not differ between the midazolam and placebo groups (69.5 [10.7] vs 69.6 [10.8], respectively; mean difference, −0.2; 95% CI, −1.9 to 1.6; *P* = .85). Sensitivity (per-protocol population, multiple imputation) and subgroup analyses (anxiety, frailty, sex, and previous surgical experience) did not alter the primary results. Secondary outcomes did not differ, except for a higher proportion of patients with hypertension (systolic blood pressure ≥160 mm Hg) at anesthesia induction in the placebo group.

**Conclusion and Relevance:**

A single low dose of oral midazolam premedication did not alter the global perioperative patient satisfaction of older patients undergoing surgery or that of patients with anxiety. These results may be affected by the low dose of oral midazolam. Further trials—including a wider population with commonplace low-dose intravenous midazolam and plasma level measurements—are needed.

**Trial Registration:**

ClinicalTrials.gov Identifier: NCT03052660

## Key Points

**Question:**

Is global perioperative patient satisfaction different in older patients administered preoperative midazolam compared with those administered placebo?

**Findings:**

In this randomized clinical trial including 616 adult patients scheduled for elective surgery with general anesthesia, the mean global index of patient satisfaction on the first postoperative day assessed by the Evaluation du Vécu de l’Anesthésie Generale questionnaire was 69.5 in the midazolam group and 69.6 in the placebo group, indicating a nonsignificant difference.

**Meaning:**

A single low-dose oral midazolam premedication did not alter the global perioperative patient satisfaction of older patients undergoing surgery.

## Teaser

This randomized clinical trial evaluates the differences in global perioperative satisfaction in patients with preoperative administration of oral midazolam compared with placebo.

## Introduction

In the perioperative setting, older patients are increasingly needing surgery. Additionally, multimorbidity, frailty, and functional dependency are common in older patients undergoing surgery.^1^

On the one hand, guidelines—mainly based on observational studies—suggest avoiding benzodiazepine premedication for older patients to prevent postoperative complications, such as delirium.^2,3^ On the other hand, recent data from a large academic health system revealed that 66% of patients aged 65 to 74 years received midazolam during anesthetic care.^4^ Postoperative patient satisfaction is a key quality indicator of perioperative care.^5,6^ Preoperative benzodiazepines are used to reduce patients’ anxiety and presumably enhance patient satisfaction. In a large randomized clinical trial (RCT) including 1062 patients, oral premedication with lorazepam did not improve self-reported patient experience. However, in this study, less than 12% of the patients were older than 65 years.^7^ The most frequently used premedication in Germany^8^ was oral midazolam, which is a short-acting benzodiazepine. Thus far, large RCTs assessing the clinical benefit of oral midazolam premedication on patient-centered outcomes in older patients are lacking. To our knowledge, the Impact of Preoperative Midazolam on Outcome of Elderly Patients (I-PROMOTE) RCT is the first multicenter RCT to evaluate the effect of oral midazolam compared with placebo in terms of global perioperative patient satisfaction in older patients.

## Methods

### Trial Design and Setting

The I-PROMOTE trial was a multicenter, double-blind, 2-arm, parallel-group, placebo-controlled RCT performed at 9 German hospitals (7 university and 2 academic hospitals). This study was approved by the Ethics Committee of the Medical Faculty of RWTH Aachen, the local ethics committees of each participating center, and the Federal Institute for Drugs and Medical Devices. Written informed consent was obtained from each patient before enrollment. I-PROMOTE was registered at ClinicalTrials.gov and is reported in accordance with the Consolidated Standards of Reporting Trials (CONSORT) reporting guideline.^9^ The study protocol was published previously^10^; the trial protocol can be found in Supplement 1, and the statistical analysis plan can be found in Supplement 2. There was no change in the methods after the trial commencement. The Center for Translational & Clinical Research, RWTH Aachen, Aachen, Germany, performed on-site data monitoring.

### Trial Participants

Legally competent patients aged 65 to 80 years scheduled for elective inpatient surgery (excluding cardiac and intracranial surgeries) of a planned duration of at least 30 minutes, with general anesthesia, with or without additional regional anesthesia, and planned extubation after surgery were eligible. The following patients were excluded from the study: (1) those who were not fluent in German; (2) those who had a history of alcohol or drug use disorder; (3) those who had severe neurological or psychiatric disorders; (4) those who were undergoing chronic benzodiazepine treatment; (5) those who were determined to not undergo general anesthesia; (6) those who had undergone repeated operations for the same reason previously; (7) those for whom the study drugs were contraindicated; (8) those who were expected to require benzodiazepine after surgery; (9) those who explicitly requested preoperative anxiolytic drugs; and (10) those who were enrolled in another RCT. A broad baseline assessment was performed, including assessment of frailty,^11^ baseline cognitive testing via the Mini-Cog test,^12^ and the Timed Up & Go test.^13^ Patient race was determined retrospectively by the principal investigators.

### Randomization and Interventions

The allocation sequence was stratified by the study center and was computer-generated (1:1 allocation ratio; block size of 6) by the Department of Medical Informatics, University Hospital Aachen, Aachen, Germany. The allocation sequence was only known to the biostatistician (A. P. K.) and the involved pharmacy of the Medical Center Johannes Gutenberg-University Mainz, Mainz, Germany, who labeled the opaque, sealed medication containers as having encapsulated midazolam, 3.75 mg, or placebo. The patients, attending anesthesiologists, and investigators were blinded to the assigned treatment. The patients received either encapsulated midazolam, 3.75 mg, or placebo orally 30 to 45 minutes before surgery. Midazolam, 3.75 mg, conforms to a dose adjustment for older patients as recommended in the summary of product characteristics of midazolam and elsewere.^14,15^ Further, the I-PROMOTE trial aimed to be a pragmatic trial reflecting clinical routine in Germany. Midazolam, 3.75 mg, relates to a halved midazolam, 7.5 mg, tablet. The patients were followed up in the operating room, in the postanesthesia care unit, or the intensive care unit (ICU), and in the ward or via telephone at postoperative days (POD) 1 and 30. Patients received additional rescue midazolam intravenously if deemed necessary by the attending anesthesiologist in the operating room. Perioperative care was managed according to the clinical routine of each participating center.

### Primary Outcome

The primary outcome was global patient satisfaction evaluated using the self-reported Evaluation du Vécu de l’Anesthésie Generale (EVAN-G) questionnaire on POD 1.^16^

### Secondary Outcomes

Key secondary end points included the impact of premedication on several patient outcomes, such as patients’ functional and cognitive recovery from baseline to POD 30 (Instrumental Activities of Daily Living [IADL] scale and Short Blessed Test [SBT])^17,18^; the impact on postoperative delirium (Confusion Assessment Method)^19,20^; extubation time; perioperative vital data, including blood pressure, heart rate, and oxygen saturation; well-being, pain, and sleeping at baseline, before anesthesia induction, 30 to 90 minutes after extubation, and on POD 1; amnesia on POD 1; the length of ICU stay, the change in EuroQoL Health-Related Quality of Life (EQ-5D-5L)^21^ from baseline to POD 30; difference in proportion of death, serious cardiac, pulmonary, and kidney complications as well as acute stroke up to POD 30; and the difference in proportion of (serious) adverse events up to POD 30 during the hospital stay.

### Statistical Analysis

Descriptive data were presented as means with SDs, medians with IQRs, and counts with frequencies. The full analysis set was used for the primary analysis of the primary end point. A linear regression model was fitted using treatment, study center, and randomization blocks as independent variables. The treatment effect was tested using an *F* test, and 95% CIs for the treatment effect estimates were calculated. A significance level of *P* < .05 was used, and all *P* values were 1-tailed. Secondary sensitivity analyses were performed using the per-protocol population and subgroup analyses by frailty status, anxiety status, sex, and surgery experience and multiple imputation to account for missing data on the primary outcome. Frailty status was defined according to Oresanya et al.^11^ Anxiety was defined as a score higher than 12 on the Amsterdam Preoperative Anxiety and Information Scale (APAIS).^22^ Postponed surgery after study treatment and postponed assessment of the primary outcome were considered major protocol deviations.

For the secondary end points, differences in means, proportions, and corresponding SEs were calculated. Adverse events and protocol deviations were analyzed in the safety population, which included all patients who received any study medication. R version 4.1.0 (The R Foundation) was used for statistical analyses.

The sample size for the study was based on the detection of a 5-unit difference in the primary outcome variable. Assuming an SD of 14 units based on previous research,^16^ 248 patients per intervention group are needed to detect a difference using type 1 and type 2 errors of .05 and .20, respectively.

## Results

Between October 2017 and May 2019 (last follow-up, June 24, 2019), 3605 patients were screened for eligibility, and a total of 616 patients were selected and randomized. Among these, 2 patients were additionally enrolled to allow parallel randomization of the last patients in 3 centers. Nine patients were excluded from the primary analysis as they had not received any study medication. Consequently, we analyzed 607 patients in the full analysis set (304 in the midazolam group and 303 in the placebo group) (Figure). Of these, 377 (62.1%) were male, and the mean (SD) age was 71.9 (4.4) years. All included patients were White of European ancestry. Baseline patient characteristics and procedural data are presented in Table 1. These did not show any differences.

**Figure.**
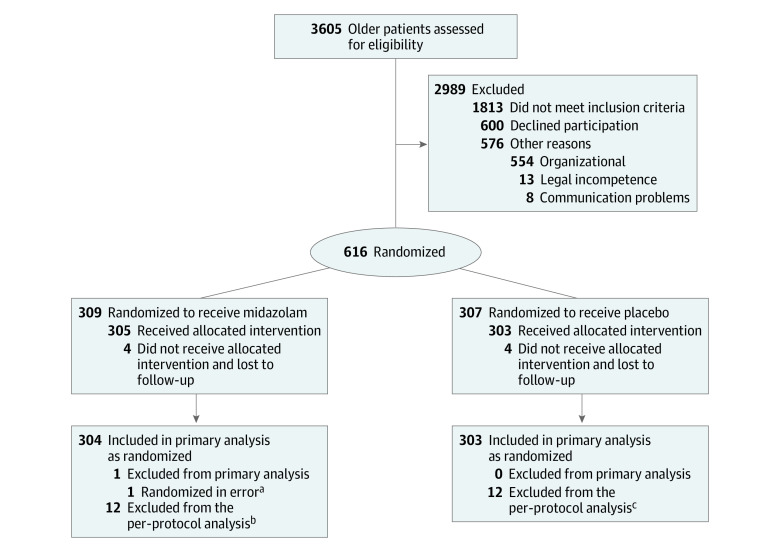
Enrollment, Randomization, and Follow-Up of Patients in the I-PROMOTE Trial ^a^Surgery was cancelled independent of the allocated intervention. ^b^Surgery was postponed to another day independent of the study intervention because of organizational reasons (n = 7), because the patient did not receive the allocated intervention (n = 4), or because assessment of the primary end point was delayed for 2 days (n = 1). ^c^Surgery was postponed to another day independent of the study intervention because of organizational reasons (n = 8), because the patient was lost to follow-up for the primary end point (n = 2), or because of a deviation from inclusion criteria (n = 2).

**Table 1.** Baseline Patient Characteristics and Procedures

Characteristic	No. (%)	
	Midazolam (n = 304)	Placebo (n = 303)
Sex		
Female	106 (35)	124 (41)
Male	198 (65)	179 (59)
Race^a^		
White of European ancestry	304 (100)	303 (100)
Age, y		
Mean (SD)	71.5 (4.4)	72.3 (4.4)
Median (IQR)	71 (68-75)	72 (69-76)
Height, cm		
Mean (SD)	171.9 (9.1)	170.5 (9.3)
Median (IQR)	172 (167-178)	170 (164-178)
Weight, kg		
Mean (SD)	80.5 (15.4)	79.2 (14.8)
Median (IQR)	80 (70-90)	80 (70-88)
BMI, mean (SD)^b^	27.2 (4.6)	27.2 (4.4)
Smoking		
Current smoker or history of smoking, No./total No. (%)	128/297 (42)	134/301 (44)
Pack years, mean (SD)^c^	28.1 (28.1)	28.7 (21.6)
Alcohol		
Alcohol consumption	129 (42)	121 (40)
Drinks per week, mean (SD)^d^	7.3 (8.9)	7.2 (7.2)
ASA category		
I	12 (4)	11 (4)
II	200 (66)	198 (65)
III	91 (30)	92 (30)
IV	1 (<1)	2 (1)
Previous surgery	254 (84)	258 (85)
Comorbidities		
Arterial hypertension	198 (65)	208 (69)
Adiposity (BMI ≥25)	168 (55)	171 (56)
Malignant disease	114 (38)	135 (45)
Hypercholesterolemia	79 (26)	81 (27)
Diabetes	62 (20)	50 (17)
Ischemic heart disease	41 (13)	34 (11)
Kidney disease	33 (11)	37 (12)
Pulmonary disease	21 (7)	25 (8)
Cerebrovascular disease	19 (6)	18 (6)
Anxiety		
APAIS score anxiety, mean (SD)^e^	8.3 (4.0)	8.8 (4.4)
APAIS score >12^e^	46 (15)	61 (20)
Functional status		
Frailty^f^	6 (2)	5 (2)
IADL, median (IQR)^g^	8 (8-8)	8 (8-8)
Timed Up & Go, mean (SD), s^h^	11.5 (6.3)	11.7 (6.1)
EQ-5D-5L		
VAS score, mean (SD)^i^	66.2 (18.6)	70.2 (18.3)
Index value, median (IQR)	0.91 (0.77-0.97)	0.92 (0.78-1.0)
Pain, VAS, median (IQR)	10 (0-46)	10 (0-50)
Sleeping quality, VAS, mean (SD)	69 (24.7)	68 (25.1)
Well-being, VAS, mean (SD)	72 (19.4)	75 (20.0)
Cognitive status		
SBT^j^		
Median (IQR)	2 (0-6)	2 (0-6)
Patients with score >0	200 (66)	207 (68)
Mini-Cog, complete test		
Total, No.	302	300
Median (IQR)^k^	4 (3-5)	4 (2-5)
Laboratory values		
Hemoglobin		
Total, No.	290	281
Mean (SD), g/dL	13.8 (1.7)	13.7 (1.7)
Hematocrit		
Total, No.	286	280
Mean (SD), %	40.4 (4.7)	40.1 (4.9)
Albumin		
Total, No.	19	21
Mean (SD), g/dL	4.4 (0.4)	4.2 (0.4)
Creatinine		
Total, No.	280	270
Mean (SD), mg/dL	1.1 (0.9)	1.1 (0.9)
Type of surgery		
Orthopedic and trauma surgery	114 (38)	95 (31)
Urology	94 (31)	91 (30)
Digestive	21 (7)	30 (10)
Ear, nose, and throat	17 (6)	22 (7)
Gynecology	13 (4)	17 (6)
Ophthalmology	11 (4)	13 (4)
Vascular surgery	11 (4)	9 (3)
Oral and maxillofacial surgery	13 (4)	7 (2)
Superficial surgery	7 (2)	10 (3)
Plastic surgery	1 (<1)	4 (1)
Other	1 (<1)	4 (1)
Severity of surgery, No./total No. (%)		
Minor	42/302 (14)	39/301 (13)
Intermediate	152/302 (50)	161/301 (53)
Major	109/302 (36)	102/301 (34)
Type of anesthesia, No./total No. (%)		
General anesthesia	244/303 (80)	252/303 (83)
Combined general and regional anesthesia	59/303 (19)	51/303 (17)
Anesthesia duration, min		
Mean (SD)^l^	181 (98.1)	178 (101.5)
Median (IQR)^l^	164 (106-248)	150 (109-234)
Surgery duration, min		
Mean (SD)^m^	119 (82.2)	120 (84.9)
Median (IQR)^m^	93 (57-170)	93 (56-170)

Abbreviations: ASA, American Society of Anesthesiology; APAIS, Amsterdam Preoperative Anxiety and Information Scale; BMI, body mass index; EQ-5D-5L, EuroQoL Health-Related Quality of Life; IADL, Instrumental Activities of Daily Living; SBT, Short Blessed Test; VAS, visual analog scale.

SI conversion factor: To convert hemoglobin to g/L, multiply by 10; albumin to g/L, multiply by 10; creatinine to μmol/L, multiply by 88.4.

^a^ Assessed retrospectively as requested during the review process. The classification was performed by the principal investigators of each participating center.

^b^ Calculated as weight in kilograms divided by height in meters squared.

^c^ Average packs calculated as 20 cigarettes per day × years of smoking.

^d^ 1 Drink was calculated as 0.25 L of beer, 0.1 L of wine, and 0.02 L of spirits.

^e^ The APAIS was used to assess preoperative anxiety. The rating consists of 4 anxiety-related items (1, 2, 4, and 5 of a total of 6 items) and is presented as a summary score of items. Items were assessed on a 5-point Likert scale, with 1 indicating not at all and 5 indicating extremely. A predefined cutoff value of an APAIS score greater than 12 was used for the German cohort, according to Berth et al.^20^

^f^ Frailty was classified as present if at least 4 of the following 6 markers were present: Mini-Cog total score of 3 points or less^12^; albumin level of 3.3 g/dL or less; 1 or more falls in the last 6 months; hematocrit level less than 35%; Timed Up & Go test greater than 15 seconds^13^; and 3 or more comorbidities present.

^g^ IADL comprises 8 categories; summary scores range from low function and dependent (0) to high function and independent (8 for women and five for men).^16^

^h^ The patient was timed while they rose from an armchair, walked 3 m, turned, walked back, and sat down again.^13^

^i^ The EQ-5D-5L included 5 categories, and self-reported health was presented on a vertical VAS.^19^

^j^ The SBT 6-Item Orientation-Memory-Concentration test had a maximum weighted error score of 24.1.^5^

^k^ Mini-Cog is a screening tool to detect cognitive impairment or dementia.^12^

^l^ Anesthesia duration was defined as the time between the first intravenous injection of medication and the time point when the attending anesthesiologist was no longer required.

^m^ Surgery duration was defined as the time between the skin incision and the last skin suture.

### Primary End Point

The mean (SD) global index of patient satisfaction assessed using EVAN-G did not differ between the midazolam and placebo groups (69.5 [10.7] vs 69.6 [10.8], respectively; mean difference, −0.2; 95% CI, −1.9 to 1.6; *P* = .85) (Table 2). The results for the 6 EVAN-G dimensions (attention, information, privacy, pain, discomfort, and waiting) were comparable in both groups.

**Table 2.** Primary and Secondary Outcome Global Patient Satisfaction

Outcome	Midazolam (n = 304)	Placebo (n = 303)	Treatment effect (95% CI)	*P* value
Primary outcome				
EVAN-G score^a^				
Global index			−0.2 (−1.9 to 1.6)	.85
Total, No.	301	301		
Mean (SD)	69.5 (10.7)	69.6 (10.8)		
Attention				
Total, No.	301	301		
Mean (SD)	63.6 (16.4)	64.7 (16.6)		
Information				
Total, No.	301	301		
Mean (SD)	61.9 (16.5)	61.2 (17.3)		
Privacy				
Total, No.	300	301		
Mean (SD)	59.4 (16.0)	60.8 (15.9)		
Pain				
Total, No.	299	301		
Mean (SD)	69.7 (15.3)	68.3 (15.5)		
Discomfort				
Total, No.	300	301		
Mean (SD)	85.5 (16.0)	86.4 (15.7)		
Waiting				
Total, No.	299	301		
Mean (SD)	77.2 (25.6)	76.3 (26.8)		
Subgroup analyses				
EVAN-G global index score^a^				
Among men				
Total, No.	198	179	−0.9 (−3.1 to 1.2)	.40
Mean (SD)	69.4 (11.1)	70.3 (11.1)		
Among women				
Total, No.	106	124	1.1 (−1.7 to 3.9)	.45
Mean (SD)	69.9 (9.9)	68.6 (10.6)		
Among patients with anxiety (APAIS score >12)^b^				
Total, No.	46	61	1.1 (−3.1 to 5.2)	.61
Mean (SD)	68.0 (7.4)	67.1 (12.5)		
Among patients with frailty^c^				
Total, No.	6	5	−0.8 (−13.6 to 11.9)	.90
Mean (SD)	63.4 (6.8)	64.7 (12.4)		
Among patients with previous surgery experience				
Total, No.	254	258	−0.4 (−2.3 to 1.4)	.64
Mean (SD)	69.4 (10.5)	69.8 (10.9)		
Among patients without major protocol deviations				
Total, No.	292	291	−0.2 (−1.9 to 1.5)	.81
Mean (SD)	69.5 (10.6)	69.7 (10.8)		

Abbreviations: APAIS, Amsterdam Preoperative Anxiety and Information Scale; EVAN-G, Evaluation du Vécu de l’Anesthésie Generale.

^a^ The EVAN-G includes 26 items within 6 dimensions.^16^ The mean score for each of the 6 dimensions (attention, privacy, information, pain, discomfort, and waiting) was calculated for each individual. If fewer than half of the items were missing, then the mean of the nonmissing items was substituted. Scores were linearly transformed to a scale of 0 to 100. The global index score is calculated as the mean dimension score.

^b^ Anxiety was measured using the APAIS. Most patients with anxiety were defined as those with an APAIS score greater than 12, according to the German cutoff level for most patients with anxiety.^20^

^c^ Patients were defined as frail if at least 4 of the following 6 markers were present: Mini-Cog total score of 3 points or less; albumin level of 3.3 g/dL or less; more than 1 fall in the last 6 months; hematocrit level of less than 35%; preoperative functional status according to the Timed Up & Go test greater than 15 seconds^13^; and 3 or more comorbidities present according to Oresanya et al.^11^

### Sensitivity and Subgroup Analyses

The sensitivity analysis with multiple imputed data—where treatment, study site, randomization block, age, sex, and American Society of Anesthesiologists physical status served as predictors—showed similar results (midazolam group, 69.5 [0.6]; placebo group, 69.6 [0.6]; mean difference, −0.1; 95% CI, −1.8 to 1.7; *P* = .90) for the primary outcome. Analysis of patients without major protocol deviations revealed no difference in treatment effect (midazolam group, 69.5 [10.6]; placebo group, 69.7 [10.8]; mean difference, −0.2; 95% CI, −1.9 to 1.5; *P* = .81) (Table 2).

Furthermore, subgroup analyses of male and female patients and patients with anxiety and frailty did not alter the global index values (Table 2). Owing to the low number of patients (n = 11) rated as frail, we performed additional, non-prespecified subgroup analyses with altered frailty definitions (eTable 1 in Supplement 3). First, we assumed that the missing albumin levels (n = 567) were 3.3 g/dL. Second, we used the Self-Care result of the EQ-5D-5L test to evaluate the preoperative functional status. In addition, we determined the cutoff value for most patients with anxiety at a score of 12 or greater, which resulted in selection of patients with greater anxiety (n = 145), but the treatment effect remained nonsignificant (eTable 1 in Supplement 3).

### Secondary Outcomes

All results of the secondary outcomes are shown in Table 3 as well as eTables 2 to 4 and eFigures 1 to 9 in Supplement 3.

**Table 3.** Secondary Outcomes

Outcome	No./total No. (%)	
	Midazolam (n = 304)	Placebo (n = 303)
At arrival in the operating room		
SpO_2_ <95%	79/302 (26)	63/303 (21)
Systolic blood pressure >160 mm Hg	83/303 (27)	140/303 (46)
Systolic blood pressure <100 mm Hg	22/304 (7)	14/303 (5)
Heart rate >100 beats/min	9/303 (3)	10/303 (3)
Patient cooperation, mean (SD), VAS^a^	96.0 (14.8)	96.0 (14.0)
Intravenous midazolam required^b^	2/304 (<0.1)	0/303
Time to extubation^c^		
Total, No.	302	299
Mean (SD), min	10.0 (6.9)	9.2 (6.3)
After extubation in the operating room		
SpO_2_ <95%	86/303 (28)	91/300 (30)
Systolic blood pressure >160 mm Hg	67/303 (22)	74/302 (24)
Systolic blood pressure <100 mm Hg	15/304 (5)	14/303 (5)
Heart rate >100 beats/min	41/290 (13)	31/290 (10)
Pain, VAS, median (IQR)		
Total, No.	280	277
Median (IQR), VAS	0 (0-20)	0 (0-15)
Well-being, VAS, mean (SD)		
Total, No.	269	262
Mean (SD), VAS	72 (24.6)	73 (23.8)
In the recovery room or ICU^d^		
SpO2 <95%	203/304 (67)	197 (65)
Piritramid^e^		
Median (IQR), mg	0 (0-6.5)	0 (0-6.0)
Patients with score >0	131/304 (43)	130/303 (43)
Pain		
Total, No.	299	296
Median (IQR), VAS	19 (0-40)	20 (0-40)
Well-being, VAS, mean (SD)		
Total, No.	289	285
Mean (SD), VAS	66 (22.9)	66 (22.2)
Outcome at postoperative day 1		
Cognitive function		
SBT^f^		
Median (IQR)	0 (0-4)	0 (0-4)
Patients with score >0	142/304 (47)	138/303 (46)
Delirium (CAM)	1/304 (<1)	3/303 (1)
Amnesia^g^	84/301 (28)	56/300 (18)
Unpleasant feeling	4/304 (13)	4/303 (13)
Postoperative recovery		
Pain, median (IQR), VAS	10 (0-30)	15 (0-40)
Sleeping quality, mean (SD), VAS	52 (30.9)	52 (29.2)
Well-being, mean (SD), VAS	72 (20.5)	72 (20.6)
Adverse events		
Adverse events (total)^h^	265/304 (87)	259/303 (85)
Hypotension	240/304 (79)	236/303 (78)
Bradycardia	63/304 (21)	66/303 (22)
Hypertension	40/304 (13)	41/303 (14)
Desaturation	27/304 (9)	24/303 (8)
Hypothermia	12/304 (4)	3/303 (1)
Tachycardia	7/304 (2)	7/303 (2)
Cardiac arrhythmia	3/304 (1)	5/303 (2)
Respiratory insufficiency	3/304 (1)	2/303 (<1)
Bronchospasm	3/304 (1)	0/303
Laryngospasm	2/304 (<1)	1/303 (<1)
Anaphylaxis	2/304 (<1)	0/303
Aspiration	1/304 (<1)	0/303
Myocardial infarction	0/304	1/303 (<1)
Cardiopulmonary resuscitation	0/304	0/303
Other events^i^	44/304 (15)	45/303 (15)
Serious adverse events	7/304 (2)	13/303 (4)
Outcomes at postoperative day 30^j^		
Death	0/298	2/297 (1)
Cardiac complication^k^	1/297 (<1)	5/293 (2)
Pulmonary complication^k^	3/297 (1)	2/293 (<1)
Stroke^k^	0/297	2/293 (<1)
Acute kidney injury^k^	2/297 (<1)	1/293 (<1)
Hospital length of stay, median (IQR)^l^		
Total, No.	301	303
Median (IQR), d	6 (3-8)	6 (3-8)
ICU length of stay^l^		
Total, No.	301	303
Median (IQR), d	0 (0-0)	0 (0-0)
Patients with score >0	25/304 (8)	33/303 (11)
Cognitive function		
SBT^f^		
Median (IQR)	0 (0-2)	0 (0-2)
Patients with score >0	113/304 (37)	122/303 (40)
Functional status		
IADL, median (IQR)^m^	8 (8-10)	8 (8-10)
EQ-5D-5L, index value, median (IQR)^n^	0.92 (0.83-0.97)	0.92 (0.84-0.97)
EQ-5D-5L, VAS score, mean (SD)^n^	71 (17.9)	73 (18.1)

Abbreviations: CAM, Confusion Assessment Method; EQ-5D-5L, EuroQoL Health-Related Quality of Life; IADL, Instrumental Activities of Daily Living; ICU, intensive care unit; SBT, Short Blessed Test; SpO_2_, oxygen saturation; VAS, visual analog scale.

^a^ Patient cooperation was rated by the attending anesthesiologist via VAS (scale of 0 to 100), with 0 indicating no cooperation to 100 indicating absolutely cooperative.

^b^ Additionally required, intravenously administered midazolam between the study treatment and the beginning of surgery.

^c^ Time to extubation was defined as the time between termination of the administration of the hypnotic agent and extubation.

^d^ Patients were transferred to the recovery room or ICU within 0.5 to 1.5 hours postoperatively.

^e^ Piritramid in total after surgery until pain on the VAS was ≤3.

^f^ The SBT 6-Item Orientation-Memory-Concentration test had a maximum weighted error score of 24.1.^5^

^g^ Amnesia was determined by patient self-assessment.

^h^ Multiple adverse events per patient was possible.

^i^ Further description of other events is presented in eTable 4 in Supplement 3.

^j^ 30-Day postoperative assessment is based on telephone interview and hospital database review.

^k^ Serious cardiac or pulmonary complications, acute stroke, or acute kidney injury at any time until day 30 after surgery. After hospital discharge, events were only rated as present if they led to readmission or death.

^l^ Hospital length of stay included the day of surgery and excluded the day of discharge. The ICU length of stay included the day of ICU admission and excluded the day of discharge from the ICU.

^m^ IADL comprises 8 categories; summary scores range from low function and dependent (0) to high function and independent (8 for women and five for men).^16^

^n^ The EQ-5D-5L included 5 categories, and self-reported health was presented on a vertical VAS.^19^

#### Day of Surgery

On the day of surgery, we found a higher proportion of patients with a systolic blood pressure greater than 160 mm Hg in the placebo group (140 of 303 [46.2%]) than in the midazolam group (83 of 303 [27.4%]). This difference did not persist after extubation. Patient cooperation was excellent in both groups before anesthesia induction, and only 2 patients in the midazolam group required additional intravenous midazolam before anesthesia induction. Time to extubation was similar.

#### Health-Related Perioperative Quality of Life

There was no difference in the visual analog scale scores for well-being, pain, sleeping, and EQ-5D-5L–rated health status at the perioperative single time points (Table 1 and Table 3) nor in the means of perioperative change in visual analog scale scores for the respective outcomes (eTable 2 and eFigures 1 to 4 in Supplement 3). The levels of problems in particular dimensions of the EQ-5D-5L are presented in eFigures 5 to 9 in Supplement 3. Both groups showed a similar distribution of responses to the EQ-5D-5L dimensions at baseline and 30-day follow-up. The distribution significantly changed for the dimensions Usual Activities, Pain/Discomfort, and Anxiety/Depression for both groups and for Self-Care in the placebo group (eTable 3 in Supplement 3). After 30 days, more patients presented with problems in the dimensions of Usual Activities and Self-Care, whereas the dimensions of Pain/Discomfort and Anxiety/Depression were less frequent.

#### Functional and Cognitive Recovery

No significant difference was found with regard to functional recovery assessed by IADL scores, and perioperative cognition remained comparable at a normal level (defined as weighted median Short Blessed Test scores less than 5) until POD 30 in both groups (Table 1 and Table 3).^17^ There was no difference in the incidence of postoperative delirium at POD 1 (Table 3). Amnesia was more frequent in the midazolam group (84 of 301 [27.9%]) than in the placebo group (56 of 300 [18.7%]) (Table 3).

#### Adverse Outcomes and Length of Stay

Neither the incidence of adverse or serious adverse events nor the incidence of predefined serious complications up to POD 30 differed between the groups (Table 3; eTable 4 in Supplement 3). The median (IQR) length of hospital stay was 6 (3-8) days in both groups; 25 of 301 patients (8%) in the midazolam and 33 of 303 (11%) in the placebo group were admitted to an ICU (Table 3).

## Discussion

We found that among older patients 65 years and older undergoing surgery, preoperative oral administration of low-dose midazolam did not alter perioperative overall patient satisfaction compared with placebo. Several secondary analyses, cohort studies, and RCTs have previously analyzed younger patients or other benzodiazepines, such as lorazepam.^1,6,7,23,24,25,26,27^ The primary result is in line with the findings of the PremedX RCT,^7^ which analyzed oral lorazepam premedication in younger patients. However, whereas patients in the PremedX trial reported higher satisfaction with lorazepam in terms of pain, discomfort, and waiting dimensions of the EVAN-G questionnaire, we could not confirm any altered effect with midazolam in this older population. Likewise, the consideration of a possible center effect^6^ in our adjusted sensitivity analysis confirmed the main results.

Even among 18% of patients with severe preoperative anxiety (APAIS score greater than 12), the treatment group did not significantly benefit from midazolam premedication regarding the index of perioperative self-reported satisfaction. This result adds new evidence that is contrary to the existing guideline, which suggests that patients with anxiety would benefit from routine premedication.^2^ Notably, the I-PROMOTE trial has not directly assessed the satisfactory anxiolysis in patients with severe anxiety.

Among the secondary outcomes, more patients in the control group arrived in the operating room with a systolic blood pressure greater than 160 mm Hg. This might indicate a higher stress level, which in turn has been shown to be associated with adverse outcomes, such as higher infection rates and increased postoperative pain and anxiety.^23^ However, in our study, postoperative pain levels up to POD 1 and adverse events up to POD 30 were similar in both groups. Notably, we did not measure perioperative stress hormones, and the I-PROMOTE trial was not powered for any secondary outcome variable.

Interestingly, extubation time was unaffected by midazolam compared with previously reported use of lorazepam.^7^ Main differences from the PremedX trial were the use of midazolam instead of lorazepam and waiving a third study group without any treatment.^7^ Besides its widespread use,^4,8,26^ midazolam is short acting, with an elimination half-life of 90 to 150 minutes.^28,29^ Thus, our mean anesthesia duration of approximately 180 minutes might explain why we did not find any substantial effect of midazolam on the postoperative outcome. Further, the applied low oral dose of midazolam, 3.75 mg, might have influenced the lack of differences compared with placebo.

As expected, amnesia was more frequent in the premedication group but was not rated as unpleasant compared with placebo. Amnesia might even be experienced beneficial by patients.^7^ Thus, amnesia might also have affected our results of patient satisfaction, for example, regarding the pain, waiting, or attention dimensions of the EVAN-G.

Recently, 3 nonrandomized studies on older patients did not find a significant association between midazolam premedication and postoperative delirium or 30-day mortality.^8,26,27^ We revealed neither different incidences of delirium limited to POD 1 nor differences in the 30-day mortality rate. Of note, the I-PROMOTE trial was not powered for these outcomes. Also, we could not confirm associations of lower incidences of postoperative nausea and vomiting in the midazolam group.^30^

The pivotal role of the self-reported health status in the perioperative setting is unquestioned.^31^ Thus far, midazolam premedication could not improve measures of health status, such as postoperative recovery up to POD 7, in younger patients.^24,32^ In addition, our results did not show improvement in the well-being, pain, and sleep quality up to POD 1 as well as in the health-related quality of life assessed by the EQ-5D-5L up to POD 30 with midazolam in older patients. Moreover, in our study, midazolam did not induce more adverse events. We also did not find any reduction in health status, such as reduced rate of early postoperative cognitive recovery, as previously described with lorazepam premedication.^8^ Notably, delayed neurocognitive recovery was previously reported after midazolam premedication.^33,34^ One reason why the I-PROMOTE trial was not able to detect differences in neurocognitive recovery might be the applied low dose of midazolam, 3.75 mg. A further reason might be that only legally competent patients without neuropsychiatric disorders were eligible, and thus patients with enhanced risk were missed. We acknowledge that there may be valid reasons that justify midazolam premedication, like improved psychological recovery along with reduced postoperative infections^23^ or prevention of explicit recall of perioperative events.^35^

### Limitations

The I-PROMOTE trial has several limitations. First, we did not analyze the plasma levels of midazolam. A lower interindividual variability of the context-sensitive half-time was shown for older patients.^28^ Thus, we considered the altered pharmacodynamics for older patients with a theoretically 50% reduced half-maximum concentration and administered only the half dose (3.75 mg) of midazolam.

Second, according to the routine in Germany, we used oral midazolam instead of intravenous midazolam, which is common practice in the US.^4^ Time to clinical effect^15^ and peak plasma concentration^36^ differ significantly between the 2 modes of midazolam intake. Thus, the time point of application was standardized to 30 to 45 minutes preoperatively, according to the summary of product characteristics.

Third, we excluded patients with cardiac, cranial, or emergency operations; all patients were White of European ancestry; and most had an American Society of Anesthesiologists status of grade II. Thus, our results may not be directly generalizable to patients with other racial or ethnic distributions, impairment, or aforementioned excluded procedures, who are frequently at high risk. In addition, exclusion of patients with severe neurological or psychiatric disorders or chronic benzodiazepine treatment might have influenced our results. These patients are at higher risk of adverse outcomes, such as postoperative delirium or 30-day mortality.^1,2^

Fourth, we could not confirm any anesthetic-sparing effect of midazolam,^24^ as we did not assess the type and dosage of the used anesthetics. Furthermore, we did not consider the influence of applied anesthetics on the primary outcome.

Fifth, 24 patients were not analyzed per protocol. However, a sensitivity analysis using the per-protocol population confirmed our primary results.

Sixth, we have not assessed whether the self-assessed patient attitudes align with actual treatment success or perioperative complications. It is known that postoperative complications and pain are associated with lower patient-reported satisfaction.^37^ Nevertheless, the assessed complications did not differ between the groups.

Seventh, we have not assessed the Hawthrone effect,^38^ where study participants tend to modify their behavior just on basis of participation or receiving a treatment, as we have not used a third comparison group without any medication. However, the previously published PremedX trial did not show any significant difference regarding the global patient satisfaction between placebo and no treatment group.^7^ Moreover, placebo interventions might influence patient-reported outcomes.^39^ Yet we are not able to draw any conclusion about a possible nocebo effect, like previously revealed elevated pain and anxiety levels in the placebo group.^7^

## Conclusions

For older patients undergoing surgery and even for patients with anxiety, a single low dose of oral midazolam, 3.75 mg, premedication did not enhance the global perioperative patient satisfaction. The other outcome indicators did not differ, except for a higher proportion of patients with hypertension at anesthesia induction in the placebo group. Our results may be affected by the low dose of oral midazolam. Nevertheless, we believe that our results can be used as a basis for a large multicenter RCT, which should consider the excluded patient population; focus on more frail patients; analyze plasma levels of midazolam, stress hormones, and inflammatory reactions; and use commonplace low-dose intravenous midazolam to confirm our results in a wider population.

## Notes

Supplement 1. Trial Protocol

Supplement 2. Statistical Analysis Plan

Supplement 3. **eTable 1.** Further Sensitivity Analyses of Primary End Point **eTable 2.** Perioperative Change of Health-Related Visual Analog Scale Score **eFigure 1.** Visual Analog Scale for Well-Being **eFigure 2.** Visual Analog Scale for Pain **eFigure 3.** Visual Analog Scale for Sleeping Quality **eFigure 4.** Visual Analog Scale for EuroQol Health **eFigure 5.** Proportion of Responses by Level Of Severity for the EQ-5D-5L Mobility Dimension at Baseline and at 30-Day Follow-Up **eFigure 6.** Proportion of Responses by Level of Severity for the EQ-5D-5L Self-Care Dimension at Baseline and at 30-Day Follow-Up **eFigure 7.** Proportion of Responses by Level of Severity for the EQ-5D-5L Usual Activity Dimension at Baseline and at 30-Day Follow-Up **eFigure 8.** Proportion of Responses by Level of Severity for the EQ-5D-5L Pain/Discomfort Dimension at Baseline and at 30-Day Follow-Up **eFigure 9.** Proportion of Responses by Level of Severity for the EQ-5D-5L Anxiety/Depression Dimension at Baseline and at 30-Day Follow-Up **eTable 3.** EQ-5D-5L Domains With Level of Problems per Point of Time **eTable 4.** Breakdown of Other Adverse Events

Supplement 4. **Group Information.** I-PROMOTE Study Group

Supplement 5. Data Sharing Statement

## References

[1] POSE-Study group. Peri-interventional outcome study in the elderly in Europe: a 30-day prospective cohort study. Eur J Anaesthesiol. 2022;39(3):198-209. doi:10.1097/EJA.0000000000001639 34799496 PMC8815832

[2] Aldecoa C, Bettelli G, Bilotta F, . European Society of Anaesthesiology evidence-based and consensus-based guideline on postoperative delirium. Eur J Anaesthesiol. 2017;34(4):192-214. doi:10.1097/EJA.0000000000000594 28187050

[3] By the 2019 American Geriatrics Society Beers Criteria® Update Expert Panel. American Geriatrics Society 2019 updated AGS Beers Criteria for potentially inappropriate medication use in older adults. J Am Geriatr Soc. 2019;67(4):674-694. doi:10.1111/jgs.15767 30693946

[4] Lei VJ, Navathe AS, Seki SM, Neuman MD. Perioperative benzodiazepine administration among older surgical patients. Br J Anaesth. 2021;127(2):e69-e71. doi:10.1016/j.bja.2021.05.016 34144785

[5] Neuman MD. Patient satisfaction and value in anesthesia care. Anesthesiology. 2011;114(5):1019-1020. doi:10.1097/ALN.0b013e318216ea25 21448059

[6] Boncyk C, Hess AS, Gaskell A, Sleigh J, Sanders RD; ConsCIOUS group. Does benzodiazepine administration affect patient satisfaction: a secondary analysis of the ConCIOUS study. Br J Anaesth. 2017;118(2):266-267. doi:10.1093/bja/aew456 28100532

[7] Maurice-Szamburski A, Auquier P, Viarre-Oreal V, ; PremedX Study Investigators. Effect of sedative premedication on patient experience after general anesthesia: a randomized clinical trial. JAMA. 2015;313(9):916-925. doi:10.1001/jama.2015.1108 25734733

[8] Stuff K, Kainz E, Kahl U, . Effect of sedative premedication with oral midazolam on postanesthesia care unit delirium in older adults: a secondary analysis following an uncontrolled before-after design. Perioper Med (Lond). 2022;11(1):18. doi:10.1186/s13741-022-00253-4 35585564 PMC9118741

[9] Moher D, Hopewell S, Schulz KF, . CONSORT 2010 explanation and elaboration: updated guidelines for reporting parallel group randomised trials. BMJ. 2010;340:c869. 20332511 10.1136/bmj.c869 PMC2844943

[10] Kowark A, Rossaint R, Keszei AP, ; I-PROMOTE study group. Impact of PReOperative Midazolam on OuTcome of Elderly patients (I-PROMOTE): study protocol for a multicentre randomised controlled trial. Trials. 2019;20(1):430. doi:10.1186/s13063-019-3512-3 31307505 PMC6632125

[11] Oresanya LB, Lyons WL, Finlayson E. Preoperative assessment of the older patient: a narrative review. JAMA. 2014;311(20):2110-2120. doi:10.1001/jama.2014.4573 24867014

[12] Alagiakrishnan K, Marrie T, Rolfson D, . Simple cognitive testing (Mini-Cog) predicts in-hospital delirium in the elderly. J Am Geriatr Soc. 2007;55(2):314-316. doi:10.1111/j.1532-5415.2007.01058.x 17302678

[13] Podsiadlo D, Richardson S. The timed “Up & Go”: a test of basic functional mobility for frail elderly persons. J Am Geriatr Soc. 1991;39(2):142-148. doi:10.1111/j.1532-5415.1991.tb01616.x 1991946

[14] Rossaint R, Werner C, Zwißler B, eds. Die Anästhesiologie. Springer-Verlag; 2012. doi:10.1007/978-3-642-21125-6

[15] Hong JC. Chapter 14 - midazolam: mechanism and perioperative applications. In: Rajendram R, Patel VB, Preedy VR, Martin CR, eds. Treatments, Mechanisms, and Adverse Reactions of Anesthetics and Analgesics. Academic Press; 2021:131-138.

[16] Auquier P, Pernoud N, Bruder N, . Development and validation of a perioperative satisfaction questionnaire. Anesthesiology. 2005;102(6):1116-1123. doi:10.1097/00000542-200506000-00010 15915023

[17] Katzman R, Brown T, Fuld P, Peck A, Schechter R, Schimmel H. Validation of a short Orientation-Memory-Concentration Test of cognitive impairment. Am J Psychiatry. 1983;140(6):734-739. doi:10.1176/ajp.140.6.734 6846631

[18] Lawton MP, Brody EM. Assessment of older people: self-maintaining and instrumental activities of daily living. Gerontologist. 1969;9(3):179-186. doi:10.1093/geront/9.3_Part_1.179 5349366

[19] Inouye SK, van Dyck CH, Alessi CA, Balkin S, Siegal AP, Horwitz RI. Clarifying confusion: the confusion assessment method. a new method for detection of delirium. Ann Intern Med. 1990;113(12):941-948. doi:10.7326/0003-4819-113-12-941 2240918

[20] Ely EW, Inouye SK, Bernard GR, . Delirium in mechanically ventilated patients: validity and reliability of the confusion assessment method for the intensive care unit (CAM-ICU). JAMA. 2001;286(21):2703-2710. doi:10.1001/jama.286.21.2703 11730446

[21] EuroQol Group. EuroQol–a new facility for the measurement of health-related quality of life. Health Policy. 1990;16(3):199-208. doi:10.1016/0168-8510(90)90421-9 10109801

[22] Berth H, Petrowski K, Balck F. The Amsterdam Preoperative Anxiety and Information Scale (APAIS)—the first trial of a German version. Psychosoc Med. Published online February 20, 2007. 19742298 PMC2736533

[23] Kain ZN, Sevarino F, Pincus S, . Attenuation of the preoperative stress response with midazolam: effects on postoperative outcomes. Anesthesiology. 2000;93(1):141-147. doi:10.1097/00000542-200007000-00024 10861157

[24] Kim MH, Kim MS, Lee JH, Seo JH, Lee JR. Can quality of recovery be enhanced by premedication with midazolam?: a prospective, randomized, double-blind study in females undergoing breast surgery. Medicine (Baltimore). 2017;96(7):e6107. doi:10.1097/MD.0000000000006107 28207530 PMC5319519

[25] Radtke FM, Franck M, Hagemann L, Seeling M, Wernecke KD, Spies CD. Risk factors for inadequate emergence after anesthesia: emergence delirium and hypoactive emergence. Minerva Anestesiol. 2010;76(6):394-403. 20473252

[26] Wang ML, Min J, Sands LP, Leung JM; the Perioperative Medicine Research Group. Midazolam premedication immediately before surgery is not associated with early postoperative delirium. Anesth Analg. 2021;133(3):765-771. doi:10.1213/ANE.0000000000005482 33721875 PMC8373629

[27] Kowark A, Berger M, Rossaint R, Schmid M, Coburn M; POSE-Study group. Association between benzodiazepine premedication and 30-day mortality rate: a propensity-score weighted analysis of the Peri-interventional Outcome Study in the Elderly (POSE). Eur J Anaesthesiol. 2022;39(3):210-218. doi:10.1097/EJA.0000000000001638 34817420 PMC8815825

[28] Albrecht S, Ihmsen H, Hering W, . The effect of age on the pharmacokinetics and pharmacodynamics of midazolam. Clin Pharmacol Ther. 1999;65(6):630-639. doi:10.1016/S0009-9236(99)90084-X 10391668

[29] Steiner C, Steurer MP, Mueller D, Zueger M, Dullenkopf A. Midazolam plasma concentration after anesthesia premedication in clinical routine—an observational study: Midazolam plasma concentration after anesthesia premedication. BMC Anesthesiol. 2016;16(1):105. doi:10.1186/s12871-016-0262-6 27776488 PMC5078941

[30] Ahn EJ, Kang H, Choi GJ, Baek CW, Jung YH, Woo YC. The effectiveness of midazolam for preventing postoperative nausea and vomiting: a systematic review and meta-analysis. Anesth Analg. 2016;122(3):664-676. doi:10.1213/ANE.0000000000001062 26516802

[31] Myles PS, Weitkamp B, Jones K, Melick J, Hensen S. Validity and reliability of a postoperative quality of recovery score: the QoR-40. Br J Anaesth. 2000;84(1):11-15. doi:10.1093/oxfordjournals.bja.a013366 10740540

[32] van Beek S, Kroon J, Rijs K, Mijderwijk HJ, Klimek M, Stolker RJ. The effect of midazolam as premedication on the quality of postoperative recovery after laparotomy: a randomized clinical trial. Can J Anaesth. 2020;67(1):32-41. doi:10.1007/s12630-019-01494-6 31576513

[33] Kahl U, Rademacher C, Harler U, . Intraoperative impaired cerebrovascular autoregulation and delayed neurocognitive recovery after major oncologic surgery: a secondary analysis of pooled data. J Clin Monit Comput. 2022;36(3):765-773. doi:10.1007/s10877-021-00706-z 33860406 PMC9162974

[34] Rajaei M, Tabari M, Soltani G, . Comparison between the effects of dexmedetomidine and midazolam on postoperative cognitive impairment after coronary artery bypasses graft surgery: a randomized clinical trial. J Tehran Heart Cent. 2019;14(2):67-73. doi:10.18502/jthc.v14i2.1374 31723348 PMC6842019

[35] Bulach R, Myles PS, Russnak M. Double-blind randomized controlled trial to determine extent of amnesia with midazolam given immediately before general anaesthesia. Br J Anaesth. 2005;94(3):300-305. doi:10.1093/bja/aei040 15567810

[36] Link B, Haschke M, Grignaschi N, . Pharmacokinetics of intravenous and oral midazolam in plasma and saliva in humans: usefulness of saliva as matrix for CYP3A phenotyping. Br J Clin Pharmacol. 2008;66(4):473-484. doi:10.1111/j.1365-2125.2008.03201.x 18537963 PMC2561102

[37] Berkowitz R, Vu J, Brummett C, Waljee J, Englesbe M, Howard R. The impact of complications and pain on patient satisfaction. Ann Surg. 2021;273(6):1127-1134. doi:10.1097/SLA.0000000000003621 31663968 PMC7303925

[38] Allen RL, Davis AS. Hawthorne effect. In: Goldstein S, Naglieri JA, eds. Encyclopedia of Child Behavior and Development. Springer; 2011:731-732. doi:10.1007/978-0-387-79061-9_1324

[39] Hróbjartsson A, Gøtzsche PC. Placebo interventions for all clinical conditions. Cochrane Database Syst Rev. 2010;2010(1):CD003974. 20091554 10.1002/14651858.CD003974.pub3 PMC7156905

