# Supplementary material for: Preoperative Midazolam and Patient-Centered Outcomes of Older Patients: The I-PROMOTE Randomized Clinical Trial
Source: JAMA Surg. 2023 Dec 20;159(2):129–38. doi: 10.1001/jamasurg.2023.6479 (PMC10733850; doi:10.1001/jamasurg.2023.6479)
Supplement: Supplement 1. — Trial Protocol [file jamasurg-e236479-s001.pdf]

Supplementary materials to

Preoperative Midazolam and Patient-Centered Outcomes of Older Patients - The I-PROMOTE Randomized Clinical Trial

Ana Kowark, MD; András P Keszei, PhD; Gerhard Schneider, MD; Stefanie Pilge, MD; Frederick Schneider, MD; David P Obert, MD; Marie-Therese Georgii, MD; Markus Heim, MD; Rolf Rossaint, MD; Sebastian Ziemann, MD; Julia van Waesberghe, MD; Michael Czaplik, PhD; Friedrich K Pühringer, MD; Christian Minarski, MD; Verena May, MD; Tobias Malisi, MD; Berthold Drexler, MD; Carmen Maria Ring, MD; Phillip Engler, MD; Roman Tilly, MD; Petra Bischoff, MD; Ulrich Frey, MD; Maria Wittmann, MD; Martin Soehle, MD; Thomas Saller, MD; Peter Kienbaum, MD; Moritz Kretzschmar, PhD; Mark Coburn*, MD; for the I-PROMOTE study group

*Corresponding author. Email: mark.coburn@ukbonn.de
I-PROMOTE-Study group. All contributing collaborators are listed in Supplement 4.

Supplement 1. Study protocol. English version including protocol changes and dates.

Protocol changes

Originally approved protocol version		Date approval by ethics committee; No.
Version 2; June 20, 2017		August 08, 2017; EK 104/17
**Protocol Version 3; April 08, 2019**	**Summary of changes**	**Change / New text**
	Change name sponsor	RWTH Aachen University<br>represented by the Rector, himself<br>represented by the Dean of the Medical Faculty
	Change name sponsor's representative	The Dean Univ.-Prof. Dr. rer. nat. Stefan Uhlig<br>Pauwelsstraße 30, 52074 Aachen, Germany<br>Te.: 0241 80 89120<br>Fax: 0241 80 82433<br>E-Mail: suhlig@ukaachen.de<br>On behalf of the Sponsor's Representative, the coordinating officer of the Center for Translational & Clinical Research Aachen (CTC-A)
	Additional information in the name for Project management	Dr. med. Ana Kowark (née Stevanovic),
	Addition of a new participating center	Klinikum rechts der Isar der Technischen Universität München<br>Klinik für Anästhesiologie<br>Ismaninger Straße 22<br>81675 München
	Change of the English wording of the study objectives	"We aim to assess, if placebo compared to preoperative administration of midazolam in elderly patients is equal in regard to the global postoperative patient satisfaction" into:<br>"We aim to assess, if placebo compared to preoperative administration of midazolam in elderly patients is different in regard to the global postoperative patient satisfaction"
	Change of the English wording of the hypothesis	"The hypothesis of the study is that global patient satisfaction after surgery in elderly patients is similar after preoperative placebo application compared to midazolam application." into:<br>"The hypothesis of the study is that global patient satisfaction after surgery in elderly patients is different after preoperative placebo application compared to midazolam application."

I-PROMOTE

IMPACT OF PREOPERATIVE MIDAZOLAM ON OUTCOME OF ELDERLY PATIENTS:

A MULTICENTRE RANDOMISED CONTROLLED TRIAL

**Sponsor**	**RWTH Aachen University**<br>represented by the Rector, himself<br>represented by the Dean of the Medical Faculty
**Sponsor's Representative**	The Dean Univ.-Prof. Dr. rer. nat. Stefan Uhlig<br>Pauwelsstraße 30, 52074 Aachen, Germany<br>Te.: 0241 80 89120<br>Fax: 0241 80 82433<br>E-Mail: suhlig@ukaachen.de<br>On behalf of the Sponsor's Representative, the coordinating officer of the Center for Translational & Clinical Research Aachen (CTC-A)<br>Dr. med. Susanne Isfort<br>Managing Director CTC-A<br>Pauwelsstraße 30, 52074 Aachen, Germany<br>T +49 241 80 80092<br>F +49 241 80 33 80092<br>E-Mail sisfort@ukaachen.de
**Sponsor`s Study Code**	16-115
**EudraCT- Number**	2016-004555-79
**Investigational Product**	Midazolam, Placebo
**Principal Coordinating Investigator**	Prof. Dr. med. Mark Coburn, MD<br>Managing Senior Physician<br>Department of Anaesthesia<br>RWTH Aachen University Hospital, Germany
**Biostatistician**	András P. Keszei, MD, PhD<br>Center for Translational & Clinical Research Aachen (CTC-A)<br>RWTH Aachen University Hospital, Germany

**Central Organisation/ Project Management**	Dr. med. Ana Kowark (née Stevanovic), MD<br>Consultant, Clinical Research Fellow<br>Department of Anaesthesia<br>RWTH Aachen University Hospital, Germany<br>T +49 241 80 35766 F +49 241 80 33 35766
**Monitoring**	Center for Translational & Clinical Research Aachen (CTC-A)<br>T +49 241 80 80092 F +49 241 80 33 80092
**Status, Version, Date of Protocol**	Final, V03, 08.04.19

Sponsor, Investigators and administrative Structure

Sponsor

The Dean Univ.-Prof. Dr. rer. nat. Stefan Uhlig

Pauwelsstraße 30, 52074 Aachen, Germany

Tel.: 0241 80 89120

Fax: 0241 80 82433

E-Mail: suhlig@ukaachen.de

on behalf of the Sponsor's Representative, the coordinating officer of the Center for Translational & Clinical Research Aachen (CTC-A)

Dr. med. Susanne Isfort

Pauwelsstr. 30, 52074 Aachen, Germany

T +49 241 80 80092

F +49 241 80 33 80092

E-MAIL SISFORT@UKAACHEN.DE Principal Coordinating Investigator

Prof. Dr. Mark Coburn
Managing Senior Physician
Department of Anaesthesia – University Hospital RWTH Aachen
Pauwelsstr. 30, D-52074 Aachen, Germany
T +49 241 80 88179
F +49 241 80 82593
E-Mail mcoburn@ukaachen.de

Investigator`s Representative and Study Coordinator

Dr. Ana Kowark (née Stevanovic)
Consultant, Clinical Research Fellow
Department of Anaesthesiology
University Hospital Aachen
Pauwelsstr. 30, 52074 Aachen, Germany
T +49 241 80 35766
F +49 241 80 3335766
E-Mail akowark@ukaachen.de

Participating Clinical Sites:

Universitätsklinikum Bonn
Klinik und Poliklinik für Anästhesiologie und Operative Intensivmedizin
Sigmund-Freud-Str. 25
53127 Bonn

Universitätsklinikum Düsseldorf
Klinik für Anästhesiologie
Gebäude 12.42
Moorenstraße 5
40225 Düsseldorf

Marienhospital Herne (Ruhr-Universität Bochum)
Klinik für Anästhesiologie, operative Intensivmedizin, Schmerz- und Palliativmedizin
Hölkeskampring 40
44625 Herne

Universitätsklinikum Magdeburg
Klinik für Anästhesiologie und Intensivtherapie
Leipzigerstr. 44
39120 Magdeburg

Klinikum der Universität München LMU
Klinik für Anästhesiologie
Marchioninistraße 15
81377 München

Klinikum rechts der Isar der Technischen Universität München
Klinik für Anästhesiologie
Ismaninger Straße 22
81675 München

Kreiskliniken Reutlingen
Klinik für Anästhesiologie und operative Intensivmedizin
Steinenbergstr. 31
72764 Reutlingen

Universitätsklinikum Tübingen
Klinik für Anästhesiologie und Intensivmedizin
Hoppe-Seyler-Straße 3
72076 Tübingen

Biostatistician

András P. Keszei, MD, PhD
Center for Translational & Clinical Research Aachen (CTC-A)
RWTH Aachen University Hospital, Germany
T +49 241 80 80355
F +49 241 80 3388790
E-mail: akeszei@mi.rwth-aachen.de

Leading Ethics Committee

Ethics Committee at the Medical Faculty of the RWTH Aachen University
Chairman: Univ.-Prof. Dr. med. G. Schmalzing
Pauwelsstraße 30, D-52074 Aachen, Germany
T +49 241 80 89963
F + 49 241 80 82012
E-Mail pprevel@ukaachen.de

Competent Authority of Germany

Federal Institute for Drugs and Medical Devices
Kurt-Georg-Kiesinger-Allee 3
53175 Bonn, Germany
T +49 228 207 4318
F +49 228 207 4355
E-Mail klinpruefung@bfarm.de

Central Organisation / Project Management

Dr. med. Ana Kowark (née Stevanovic), MD
Consultant, Clinical Research Fellow
Department of Anaesthesia
RWTH Aachen University Hospital, Germany
T +49 241 80 35766
F +49 241 80 33 35766

Monitoring

Center for Translational & Clinical Research Aachen (CTC-A)
RWTH Aachen University
Pauwelsstraße 30
52074 Aachen
T +49 241 80 80092
F +49 241 80 3380092

Table of Contents

Sponsor, Investigators and administrative Structure 3

SYNOPSIS 10

Abbreviations 16

1 STUDY RATIONALE AND CLINICAL RELEVANCE 17

1.1 Description of evidence and medical need 17

1.2 Rationale and Clinical Evidence 17

2 Aim of Study 18

2.1 Primary endpoint 18

2.2 Secondary endpoints 18

2.3 Hypothesis 19

3 INVESTIGATIONAL DRUG AND COMPARATOR 20

3.1 Investigational Drug 20

3.1.1 Packaging, Labelling and re-supply of Investigational Product (IP) 20

3.1.2 Management of IMP 20

3.1.3 Advice for using the IMP 20

3.2 Comparator 20

3.2.1 Packaging, Labelling and re-supply of the Comparator Product 20

3.3 Benefit Risk Assessment 21

4 Study Design and Duration 21

4.1 Study Design 21

4.2 Study Duration 21

5 Study Population 21

5.1 Number of Patients 21

5.2 Inclusion Criteria 21

5.3 Exclusion Criteria 22

5.4 Subjects of Reproductive Potential 23

6 Study Treatments 24

6.1 Randomisation 24

6.1.1 Randomisation procedure 24

6.1.2 Randomisation number 24

6.2 Recruitment 24

6.3 Overview Study Treatment 25

6.4 Blinding 26

6.5 Un-blinding Procedures 26

7 Treatment Details 26

7.1 Variables 26

7.1.1 Primary outcome measure: 27

7.1.2 Baseline data and secondary outcome measures: 27

8 Safety Data Collection, Recording and Reporting 31

8.1 Definition of Adverse Events (AEs) 31

8.1.1 Instructions for completing adverse event case report forms 31

8.2 Definition of Serious Adverse Events (SAEs) 33
8.3 Reporting Procedures of serious adverse events SAEs 33
9 Study Termination 33
10 Emergency Procedures 34
10.1 Emergency Contact Address/Safety Monitor 34
11 Statistics 34
12 Ethical and Legal Aspects 35
12.1 Independent Ethics Committees 35
12.2 Authorisation of the Competent Authority 35
12.3 Notification Requirements 35
12.4 Informed Consent 36
12.4.1 Post-study treatment 36
12.4.2 Subject privacy 36
12.4.3 Contact point 37
12.5 Duties of the Investigator 37
12.6 Delegation of Investigator’s Duties 37
12.7 Protocol Changes 37
12.8 Data Protection 37
13 Data Quality Assurance 38
13.1 Quality control 38
13.2 Source documentation requirements 38

13.3 Data management 39

13.4 Direct Access to Source Data 39

13.5 Monitoring 39

13.6 Auditing 40

14 Data Handling and Record Keeping 40

14.1 Conclusion of Documentation 40

14.2 Corrections to data 40

14.3 Record keeping 40

14.4 Archiving of Documents 41

14.5 Destruction of study documents 41

15 Publication Policy 41

16 Finance and Insurance 42

16.1 Financing 42

16.2 Insurance 42

17 Statement of compliance 43

18 Signatures 44

19 References 46

20 FIGURE 1 48

SYNOPSIS

Item	Description
**Study Title**	Impact of Midazolam on Perioperative Outcome of Elderly patients: a multicentre randomised controlled trial
**Study Short Name**	I-PROMOTE
**Study Number**	16-115
**EudraCT-Number**	2016-004555-79
**Phase**	IV
**Protocol version**	Version 02, 20.06.2017
**Registration with ClinicalTrials.gov**	NCT03052660
**Regulations**	In accordance to the Declaration of Helsinki, ICH E6 Guideline for Good Clinical Practice (GCP), German Medicine Act (AMG), Good Clinical Practice Act (GCP- V), Local Rules, regulations and applicable requirements
**Sponsor**	Uniklinik RWTH Aachen vertreten durch den Rektor,<br>vertreten durch den Dekan der Medizinischen Fakultät<br>Univ.-Prof. Dr. rer. nat. Stefan Uhlig<br><br>und im Auftrag des Sponsorvertreters, die Koordinierende Geschäftsführerin des Center for Translational & Clinical Research Aachen (CTC-A)<br>Dr. med. Susanne Isfort“Phone: +49 241 80 80092<br>Fax +49 241 80 33 80092<br>E-Mail: sisfort@ukaachen.de
**Investigator**	Principal Coordinating Investigator<br>Prof. Dr. med. Mark Coburn<br>Department of Anaesthesia<br>RWTH Aachen University Hospital<br>Pauwelsstraße 30,<br>52074 Aachen, Germany<br>Phone:+49 241 80 88179<br>Fax: +49 241 80 82593<br>E-Mail: mcoburn@ukaachen.de
**Financing**	This is an investigator-initiated trial. This trial will be supported by the Department of Anaesthesiology, University Hospital of RWTH Aachen, Germany

Item	Description
**Insurance**	A separate patient`s insurance is not completed in accordance with §40 (1b) of the German Medicines Act (AMG). In the case of fault-based incidences the patients are insured by the general liability insurance of the respective hospital.
**Risk Benefit Assessment**	Midazolam is a preoperatively, routinely used medication in surgical patients. Additional harms, other than the usually present side effects in the clinical routine are not expected in the midazolam-group in this study. For the placebo-group, we do not expect any harm, as in the case of strong preoperative anxiety or agitation, midazolam application may occur. If, this study shows an equal patient satisfaction and less perioperative adverse events in the placebo-group, the patients would benefit from waiving of unnecessary routinely applied premedication.
**Key Words**	Midazolam, Placebo, Premedication, Preoperative, Elderly patients
**Study Drug Trade Name ®**	Dormicum®<br>Dose: 3.75 mg<br>Mode of administration: oral
**Study Drug INN**	Midazolam
**Comparator Drug(s) INN if applicable (International Nonproprietary Name)**	Placebo<br><br>Placebo:<br>Dose: N/A<br>Mode of administration: oral
**Indication**	Midazolam: medication with anxiolytic, amnestic, hypnotic and anticonvulsant activity. Routinely used as premedication in surgical patients.
**Medical Study Rationale**	Generalised premedication with benzodiazepines in all surgical patients has become questionable, regarding the risk-benefit assessment and the lack of evidence for this practice. Particularly, in elderly patients (≥65 years), a higher risk for adverse events is described. A recently conducted randomised, placebo-controlled study in France including 1062 elective surgical patients <70 years (mean 50 years) showed no difference in regard to the patient satisfaction between three groups (2.5 mg lorazepam, placebo and no-premedication). Data for elderly patients are lacking and large

Item	Description
	randomised controlled trials are needed. Actually, many German hospitals already withheld benzodiazepine premedication from elderly patients, notwithstanding the insufficient evidence regarding this issue.
**Study Objectives**	We aim to assess, if placebo compared to preoperative administration of midazolam in elderly patients is different in regard to the global postoperative patient satisfaction
**Evaluation Criteria**	**Primary endpoint**<br>Global patient satisfaction on the first postoperative day<br>**Secondary endpoints**<br>• Assessment of preoperative anxiety<br>• Functional and cognitive recovery<br>• Postoperative delirium<br>• Perioperative condition of well-being, pain and sleeping<br>• Patient cooperation directly preoperatively<br>• Amount of patients with rescue-midazolam application<br>• Time to extubation<br>• Health-related quality of life assessment before and 30 days after surgery<br>• Longer-term outcome after 30 days including mortality and the new-onset of serious cardiac or pulmonary complications, acute stroke, or acute kidney injury<br>• Subgroup analysis according to the preoperative anxiety level, the patient demographics and surgery experience<br>• Adverse events (AEs) und serious adverse events (SAEs)<br>• Gender effects on patient satisfaction<br>• Hospital length of stay (LOS) and ICU-LOS
**Study Design**	Multicentre, randomised, placebo-controlled, double-blinded, two-arm parallel, interventional clinical trial

Item	Description
**Study Duration**	Duration of subject participation: 30 days<br>From anaesthesia-induction until the 30th postoperative day<br>In total 24 months including evaluation and clinical study report. The recruitment period will last 18 months, and data cleaning, processing, analysis and reporting 6 months as well.
**Patients Number**	614 in total (3.75 mg midazolam (n=307) and placebo (n=307))
**Inclusion Criteria**	1. Only legally competent patients<br>2. Written informed consent prior to study participation<br>3. 65-80 years<br>4. Elective surgery<br>5. Expected surgery duration ≥ 30 minutes<br>6. Planned general or combined regional and general anaesthesia<br>7. Planned extubation at the end of surgery
**Exclusion Criteria**	1. Age > 80 years<br>2. Age < 65 years<br>3. Non-fluency in German language<br>4. Alcohol and/ or drugs abuse<br>5. Chronic benzodiazepine treatment<br>6. Intracranial surgery<br>7. Local and stand by anaesthesia or solely regional anaesthesia<br>8. Monitored anaesthesia care<br>9. Cardiac surgery<br>10. Ambulatory surgery<br>11. Repeated surgery<br>12. Contraindications for benzodiazepine application (e.g. sleep apnoea syndrome, severe chronic obstructive pulmonary disease, allergy)<br>13. Allergy against any component of the Placebo (lactose monohydrate, cellulose powder, magnesium stearate, microcrystalline cellulose) or investigational drug (midazolam,

Item	Description
	lactose) or the capsules (gelatine, E171 titanium dioxide, E132 indigotine).<br>14. Expected benzodiazepine requirement after surgery<br>15. Expected continuous mandatory ventilation after surgery<br>16. Patients who explicitly request anxiolytic premedication<br>17. Patients with severe neurological or psychiatric disorders<br>18. Refusal of study participation by the patient<br>19. Parallel participation in interventional clinical studies within the last 30 days
**Treatment and Visits**	Patients, meeting all inclusion and none exclusion criteria, will be randomly assigned to either receive 3.75 mg midazolam or placebo.<br><br>**Visit 0 (Baseline Visit)**<br><br>Patient information and written informed consent. Assessment of the patient demographics, medical history, laboratory values done in the clinical routine and study-specific baseline tests (anxiety, cognitive and functional assessment, delirium assessment, health-related quality of life assessment, pain, sleeping and well-being, frailty assessment). Patient randomisation.<br><br>**Visit 1 (Surgery day, preoperative)**<br><br>Eligible and enrolled patients will receive 30-45 minutes before surgery the respective container including the allocated treatment by the investigator. In case of apparent or verbally expressed anxiety, additional rescue midazolam i.v. will be permitted in both groups.<br><br>**Visit 2 (Surgery day, intraoperative)**<br><br>Anaesthesia will be conducted according to the clinical routine. Intraoperative surgery- and anaesthesia-related data will be assessed. The attending anaesthetist will measure the time until extubation respectively removing of the airway device after cessation of the anaesthetic (inhalative or intravenous).

Item	Description
	**Visit 3 (Surgery day, postoperative)**<br>The patient will undergo further study-specific assessments in the post-anaesthesia care unit (PACU) or intensive care unit (ICU).<br>**Visit 4 (First postoperative day)**<br>A follow-up visit with study-specific assessments will be performed on the ward or ICU.<br>**Visit 5 (30. postoperative day)**<br>A follow-up visit with study-specific assessments will be performed via telephone or on ward, if the patient is still in hospital.<br>The study participation ends after the follow-up call via telephone/ visit and the hospital database review on the 30st postoperative day.
**Sample size and Statistics**	Primary analysis of the study outcome will be performed according to the intention-to-treat (ITT) principle.<br><br>Descriptive analysis of all study data will be performed for both treatment arms. Frequencies for categorical variables and means, medians, standard deviations and selected quantiles for quantitative variables will be tabulated. Distributions of variables will be graphically examined.<br><br>The primary outcome will be analysed with the use of analysis of variance (ANOVA) including treatment effect and study centre as factors. The treatment effect will be tested using an F-test.<br><br>The sample size was calculated based on detecting a minimum of 5 unit difference in the primary outcome variable overall patient satisfaction measured with the EVAN-G. Assumptions regarding the standard deviation of EVAN-G in the population was based on previous work [1]. Setting a type 1 error of 0.05, a power of 0.8 and assuming the standard deviation of EVAN-G to be 14 units, 248 patients per group are needed to detect a 5 unit difference.<br><br>Considering a drop-out of 10% and a screening failure of 10%, we decided to include 614 patients in total.

Abbreviations

AE	Adverse event
AMG	German Medicine Act
APAIS	Amsterdam Preoperative Anxiety and Information Scale
ASA	American Society of Anesthesiologists
BfArM	Federal Institute for Drugs and Medical Devices
BMI	Body mass index
CAM	Confusion Assessment Method
CRF	Case Report Form
CTC-A	Center for Translational & Clinical Research Aachen
eCRF	Electronic Case Report Form
GCP	Good Clinical Practice
GCP-V	Good Clinical Practice Act
IADL	Instrumental activities of daily living
ICH	International Declaration of Helsinki
ICU	Intensive care unit
IEC/IRB	Independent Ethics Committee/ Independent Review Board
ITT	Intention to treat
IMP	Investigational Medicinal Product
IP	Investigational Product
LOS	Length of stay
MACCE	Major adverse cardiovascular and cerebral events
PACU	Post anaesthesia care unit
POD	Postoperative delirium
PP	Per protocol
RWTH	Rheinisch-Westfälische Technische Hochschule
SAE	Serious Adverse Event
SmPC	Summary of Medicinal Products Characterisation
SOP	Standard Operation Procedure
VAS	Visual analogue scale

1 STUDY RATIONALE AND CLINICAL RELEVANCE

1.1 Description of evidence and medical need

Preoperative application of anxiolytic medication is frequently used to enhance the patient satisfaction, although we lack of clear evidence for this generalised drug administration in all surgical patients. Preoperative anxiety is multi-factorial caused and has individually different influence on the perioperative outcome [2]. Cognitive and behavioural changes, physiological reactions, different requirement for anaesthetic drugs and perception of pain, mood swings, wound-healing problems and alteration of the immune system were reported [3]. The effect on patient satisfaction and the generalised necessity for all patients is questionable in regard to the risk-benefit assessment of benzodiazepine application. Dose-dependent sedation up to respiratory depression is possible. Further, paradox reactions and the antegrade amnesia are experienced as unpleasant by some patients [4,5]. Furthermore, incidence of pneumonia with an increased mortality was associated with the intake of benzodiazepines [6,7]. Postoperative delirium (POD) in elderly patients (> 65 years), as one type of cognitive impairment, is a serious disease with frequently lethal consequences. 13-50% of the non-cardiac surgical patients experience POD [8]. The reasons are multifactorial [9], but 30-40% of the POD cases could be avoided by preventive measures. These include the avoidance of benzodiazepines, as they potentially enhance and prolong a POD and cognitive dysfunction [8,10]. In contrast, preoperative anxiety in elderly patients (> 65 years) is not associated with an increased risk for POD [11]. A non-pharmacological treatment of preoperative sleeping disorders and anxiety is recommended in these patients [8]. This is underlined in the American guideline for POD in elderly patients, which advises to avoid delirium-causing drugs including benzodiazepines [12].

If the application of premedication does not provide a significant benefit for the elderly surgical patients, waving of premedication would have a big impact on the individual patient and our health care system.

1.2 Rationale and Clinical Evidence

A recently conducted randomised, placebo-controlled study in France including 1062 elective surgical patients < 70 years (mean 50 years) showed no difference in regard to the patient satisfaction between three groups (2.5 mg lorazepam, placebo and no-premedication) [13]. The time until extubation and the early postoperative recovery were significantly prolonged respectively worse in the lorazepam group than in the control- or placebo-group. Only 24 % of the patients showed an increased preoperative anxiety level and the subgroup analysis of

these patients did not reveal a difference in regard to the overall patient satisfaction. Data for elderly patients remain lacking.

A Cochrane analysis of the anxiolytic premedication effect on time to discharge in a day case surgery setting found similar discharge times between patients with premedication compared to the placebo group, though impaired psychomotor function after benzodiazepines application [14]. Of note, this Cochrane analysis failed to report outcomes of efficacy of anxiolytic premedication and the included studies were of poor quality and very heterogeneous. Therefore a balanced judgement about risk and benefit of premedication was hindered. Another Cochrane review showed that there is a lack of evidence for premedication effects in elderly patients [9].

Currently in Germany, preoperative application of benzodiazepines in elderly patients is an important subject of controversial discussions. Actually, many hospitals already withheld benzodiazepine premedication from elderly patients, notwithstanding the insufficient evidence regarding this issue. A large randomised controlled trial (RCT) is indicated, to clarify the evidence for or against the preoperative benzodiazepine application in elderly patients.

2 Aim of Study

We aim to assess, if preoperative application of placebo compared to midazolam in elderly patients is different with regard to the global postoperative patient satisfaction.

2.1 Primary endpoint

Global patient satisfaction on the first postoperative day

2.2 Secondary endpoints

- Assessment of preoperative anxiety
- Assessment of preoperative frailty
- Functional and cognitive recovery
- Postoperative delirium
- Perioperative condition of well-being, pain and sleeping
- Patient cooperation directly preoperatively
- Amount of patients with rescue midazolam application
- Time to extubation
- Health-related quality of life assessment before and 30 days after surgery
- Longer-term outcome after 30 days including mortality or the new-onset of serious cardiac or pulmonary complications, acute stroke, or acute kidney injury

- Subgroup analysis according to the preoperative anxiety level, the patient demographics and surgery experience
- Adverse events (AEs) and serious adverse events (SAEs) according to the medical charts
- Gender effects on the primary outcome
- Hospital length of stay (LOS) and intensive care unit (ICU)-LOS

2.3 Hypothesis

The hypothesis of the study is that global patient satisfaction after surgery in elderly patients is different after preoperative placebo application compared to midazolam application.

3 INVESTIGATIONAL DRUG AND COMPARATOR

3.1 Investigational Drug

Midazolam (Dormicum®) is an approved oral or intravenous drug, which is routinely used as premedication before surgery. It incorporates anxiolytic, amnestic, hypnotic and anticonvulsant activity. The dosage of 3.75mg midazolam (halved 7.5mg tablet) was chosen according to the recommendation to reduce the dosage for elderly patients, which is described in the SmPC. Furthermore, it complies with the clinical routine in many German hospitals (including the participating centres) to use this reduced dosage in elderly patients.

The detailed information in the Summary of medicinal product characteristics (SmPC; instruction of use) will be submitted.

3.1.1 Packaging, Labelling and re-supply of Investigational Product (IP)

The investigational product midazolam will be relabelled and encapsulated by the Department of Pharmacy, University Medical Center Johannes Gutenberg-University Mainz, Germany according to MHRA [Medicines wand Healthcare Products Regulatory Agency] guidelines and provided to the investigator

3.1.2 Management of IMP

According to the clinical routine, the patients will receive the drug 30-45 minutes before the surgery.

3.1.3 Advice for using the IMP

Storage conditions: See instruction of use (SmPC).

3.2 Comparator

3.2.1 Packaging, Labelling and re-supply of the Comparator Product

The Placebo will be relabelled and supplied by the Department of Pharmacy, University Medical Center Johannes Gutenberg-University Mainz, Germany to the investigator. It will be stored according to the advice of the company.

3.3 Benefit Risk Assessment

Midazolam is a preoperatively, routinely used medication in surgical patients, however there is no medical evidence, that general application is advantageous for all patients, especially the elderly ones. Midazolam incorporates several side effects, which probably jeopardise the patient. Additional harms, other than the usually present side effects in the clinical routine are not expected in the midazolam-group in this study. All possible side effects are described in the SmPC for midazolam. For the placebo-group, we do not expect any harm, as in the case of strong preoperative anxiety or agitation, additional midazolam application may occur on behalf of the attending anaesthetist at any time. If, this study shows the same or even higher patient satisfaction, with concurrently less perioperative adverse events in the placebo-group, the individual patients would benefit from waiving of unnecessary sedative premedication administration. Furthermore, faster patient recovery and discharge times would enhance the patient comfort and improve cost-effectiveness.

4 Study Design and Duration

4.1 Study Design

This is a multicentre, controlled, double-blinded, randomised, two-arm, placebo-controlled, interventional clinical study.

4.2 Study Duration

Patient recruitment will be started in August 2017. Last patient in/ out is anticipated for December 2018 First results will be available in August 2019. The total study duration will be 24 months including evaluation and clinical study report.

5 Study Population

5.1 Number of Patients

We will enrol 614 patients in total (3.75 mg midazolam n=307 and placebo n=307), including the assumption of 10% drop-outs and 10% screening failures.

5.2 Inclusion Criteria

Subjects, fulfilling the following inclusion criteria are suitable for participation in the study:

1. Only legally competent patients
2. Written informed consent prior to study participation

3. 65-80 years, both genders
4. Elective surgery
5. Expected surgery duration ≥ 30 minutes
6. Planned general or combined regional and general anaesthesia
7. Planned extubation at the end of surgery

5.3 Exclusion Criteria

Subjects, fulfilling one or more of the following exclusion criteria will not be included in the study:

1. Age > 80 years
2. Age < 65 years
3. Non-fluency in German language
4. Alcohol and/ or drug abuse
5. Chronic benzodiazepine treatment
6. Intracranial surgery
7. Local and stand by anaesthesia or solely regional anaesthesia
8. Monitored anaesthesia care
9. Cardiac surgery
10. Ambulatory surgery
11. Repeated surgery
12. Contraindications for benzodiazepine application (e.g. sleep apnoea syndrome, severe chronic obstructive pulmonary disease, allergy)
13. Allergy against any component of the Placebo (lactose monohydrate, cellulose powder, magnesium stearate, microcrystalline cellulose) or investigational drug (midazolam, lactose) or the capsules (gelatine, E171 titanium dioxide, E132 indigotine).
14. Expected benzodiazepine requirement after surgery
15. Expected continuous mandatory ventilation after surgery
16. Patients who explicitly request anxiolytic premedication
17. Patients with severe neurological or psychiatric disorders
18. Refusal of study participation by the patient
19. Parallel participation in interventional clinical studies within the last 30 days

5.4 Subjects of Reproductive Potential

Not applicable

6 Study Treatments

6.1 Randomisation

To guarantee adequate sequence generation and allocation concealment, randomisation will be centralised and computer-based. The randomisation will be carried out by the Department of Medicinal Informatics RWTH Aachen University Hospital. A study centre stratified randomisation will be implemented. A unique randomisation number will be assigned to each randomised patient. Allocations will be made available to the pharmacy through the central Study Management Tool. The tool is accessible via a web browser based interface. The Department of Pharmacy, University Medical Center Johannes Gutenberg-University Mainz, Germany will provide sealed, opaque containers containing the assigned treatment to each centre. These containers will be marked with the ascending randomisation number.

6.1.1 Randomisation procedure

Patients, meeting all inclusion and none exclusion criteria, will be randomly assigned to either receive 3.75 mg midazolam or placebo. After written informed consent, the respective container according to the randomisation number will be handed over to an independent nurse, not involved in the study.

Access to randomisation-list is reserved solely to the biostatistician, the CTC-A and the pharmaceutics company. For emergency cases, all centres will receive opaque, sealed emergency envelopes including the information about the assigned treatment.

6.1.2 Randomisation number

The randomisation number will be consecutive and ascending for each centre. Each patient will receive a unique number, consistent of 3 digits (indicating the centre) and 3 further digits for the ascending patient number (e.g. 002-063). 002 would indicate the specific centre and 063 the patient number 63 in this centre. This randomisation code will be used to label all collected study data.

6.2 Recruitment

Patients will be recruited consecutively during the preoperative anaesthesia consultation in the clinical routine. Each participating centre will recruit as many patients as possible. Documentation of the time-point of informed consent will be required, to enable reproducing the sequence of patient recruitment and randomisation, in order to prevent selection bias. All screened patients (including the screening failures and enrolled patients) will have to be

documented in a screening/ enrollment log. The screening number will be coded independently from the randomization number with 3 digits.

6.3 Overview Study Treatment

All visits are presented in figure 1 according to the SPIRIT Statement.

Visit 0 (Baseline Visit)

After study-specific patient information and written informed consent (during the usual pre-anaesthesia visit and patient information in the clinical routine), the investigator will perform a baseline visit, which includes the assessment of the patient demographics, medical history and the most recent preoperative routine laboratory values (only if done in the clinical routine). Furthermore, the study-specific baseline testing (anxiety, cognitive and functional assessment, health-related quality of life assessment, pain, sleeping and well-being) and frailty assessment will be performed. The patient will receive the next consecutive randomisation number and the ward-staff will be informed, that the patient should not receive any benzodiazepine in the clinical routine, if not indispensable until the surgery.

Visit 1 (Surgery day, preoperative)

Eligible and enrolled patients will receive 30-45 minutes before surgery either the respective container including the allocated treatment (relabelled concealed capsule including midazolam or placebo). In case of apparent or verbally expressed anxiety, the patients will receive additional midazolam i.v., when entering the surgery area, according to the clinical routine (study- and group-independent). This midazolam will be applied carefully titrated (á 0.5mg) i.v., according to the SOP of the respective department, by the attending anaesthetist under monitoring of the patiens` vital data. Therefore, un-blinding will not be required.

Visit 2 (Surgery day, intraoperative)

Anaesthesia will be conducted according to the clinical routine, these includes also the kind of anaesthesia. Intraoperative surgery- and anaesthesia-related data will be assessed. An additional application of benzodiazepines is not desired, but left to the discretion of the attending anaesthetist, who will be blinded to the allocation treatment. The attending anaesthetist will measure the time until extubation respectively removing of the airway device after cessation of the anaesthetic agent (inhalative or intravenous).

Visit 3 (Surgery day, postoperative)

The patient will undergo further study-specific assessments in the post-anaesthesia care unit (PACU) or ICU.

Visit 4 (First postoperative day)

A follow-up visit with study-specific assessments will be performed on the ward or ICU.

Visit 5 (30. postoperative day)

A follow-up visit with study-specific assessments will be performed via telephone or visit on ward, if the patient is still in hospital. The hospital LOS and ICU-LOS data will be collected from the hospital database.

6.4 Blinding

This trial is planned in a double-blinded manner. The investigator, the intraoperative attending anaesthetist and the patient will not be aware of the treatment allocation in all cases.

6.5 Un-blinding Procedures

In the event of medical emergency, which requires identification of an individual patient's treatment, the investigators are permitted to open the respective emergency envelope. The reason must be documented in the patient`s medical record and in the eCRF, and must explain why revealing the treatment assignment was necessary to guide subsequent intervention and therapy. Un-blinding is not necessary in case of additional preoperative midazolam treatment under controlled conditions in the clinical routine (see visit 1).

7 Treatment Details

7.1 Variables

The primary aim of this study is to assess if the perioperative overall patient satisfaction is different among elderly patients with preoperative application of placebo compared to the preoperative application of midazolam. And secondary, if there is a difference in regard to the perioperative outcomes between the two study groups.

The case report form (CRF) is attached in the Appendix 1. It includes all visits and the respective outcome measurements/ study-specific tests.

7.1.1 Primary outcome measure:

Global patient satisfaction measured with the EVAN-G questionnaire [1] on the first postoperative day, at visit 4 (9 minutes).

7.1.2 Baseline data and secondary outcome measures:

Visit 0

- Patient demographics (age, gender, weight, height, body mass index (BMI), smoking status, American Society of Anaesthesiologists (ASA) physical status)
- Pre-existing diseases and medical/ surgical history
- Most recent preoperative routine laboratory values (only if done in the clinical routine): haemoglobin and haematocrit level; serum creatinine and serum albumin
- Study-specific testing: baseline assessment prior to surgery:
    1. Short blessed test (SBT) (5-10 minutes) [15]
    2. Confusion Assessment Method (CAM) (1-3 minutes) [16]
    3. Amsterdam Preoperative Anxiety and Information Scale (APAIS) (1-2 minutes) [17]
    4. Health-related quality of life assessment EQ-5D-5L (EuroQuol Group) (2-5 minutes) [18]
    5. Instrumental Activities of Daily Living (IADL) scale (2-5 minutes) [19]
    6. Visual analogue scale (VAS) pain, sleeping quality and quality of well-being (1-2 minutes).
- Frailty assessment according to Oresanya et al. [20]. This includes in addition to the medical history and laboratory values, history of falls, the Mini-Cog [21] (3 minutes) and timed "Up & Go" test [22].

Visit 1

Randomization

Visit 2

- Patient cooperation rated by the attending anaesthetist (via VAS)
- Anaesthesia and surgery-related data
    1. Drugs and kind of general anaesthesia
    2. Kind of regional anaesthesia

3. Durations (anaesthesia/ surgery/ time to extubation)
4. Kind of surgery
5. Severity of surgery
6. Rescue benzodiazepine application
7. Patients vital data:

- Upon arrival in the operating room:-
    a) Peripheral oxygen saturation (SpO_2) <95% with air
    b) Systolic blood pressure >160mmHg
    c) Systolic blood pressure <100mmHg
    d) Heart rate >100 bpm

- Until leaving the operating room after extubation:
    a) SpO_2 <95% with air
    b) Heart rate >100 bpm
    c) Systolic blood pressure <100 mmHg
- VAS pain and quality of well-being directly after end of anaesthesia (1 minute)
- AEs and SAEs according to the medical charts

Visit 3

- Study-specific testing within 0.5-1.5 hours after surgery in PACU and ICU:
    a. SpO_2 < 95% with air at any time until 1.5 hours after surgery
    b. VAS pain and quality of well-being 0.5-1.5 hours later (1 minute).
    c. AEs and SAEs according to the medical charts

Visit 4

- Study-specific testing on the first postoperative day:

a. EVAN-G (9 minutes)

b. SBT, (5-10 minutes)

c. CAM or CAM-ICU for patients on the intensive care unit (ICU), (1-5 minutes)

d. VAS pain, sleeping quality and quality of well-being, (1-2 minutes)

e. AEs and SAEs according to the medical charts

Visit 5

- **Study-specific follow-up on the 30th postoperative day (via telephone interview (if discharged)/ visit (if still in hospital) and hospital database review)**
  1. Mortality until day 30 postoperative
  2. EQ-5D-5L (2-5 minutes)
- Analysis of the new-onset of serious cardiac or pulmonary complications, acute stroke, or acute kidney injury up to 30-days after surgery (according to the following definition:)
  1. ***Serious cardiac complication*** (Cardiac arrest: The absence of cardiac rhythm or presence of a chaotic cardiac rhythm requiring the initiation of CPR, which includes chest compressions. Myocardial infarction: Electrocardiography (ECG) changes, new elevation in troponin, or physician diagnosis. Signs of myocardial infarction in the autopsy.)
  2. ***Serious pulmonary complication*** (Pneumonia: Clinical or radiological diagnosis. Pulmonary embolism: Radiological diagnosis. Signs of pneumonia or pulmonary embolism in the autopsy)
  3. ***Acute Stroke*** (Defined as a new focal or generalised neurological deficit of >24h duration in motor, sensory, or coordination functions with compatible brain imaging and confirmed by a neurologist. Transient ischemic attack is not considered as acute stroke. Signs of stroke in the autopsy.)
  4. ***Acute kidney injury*** (Defined according to the AKIN classification [23] as AKI stage ≥2. This means increase of creatinine >2-3x from baseline within the hospital stay. Or urine output less than 0.5 ml kg^{-1} per hour for more than 12 hours. Or signs of acute kidney injury in the autopsy.)

After hospital discharge, events will only be defined as present if they led to hospital re-admission or death.

3. IADL (2-5 minutes)
4. SBT (5-10 minutes)

5. Hospital LOS and ICU-LOS data collection from the hospital database.

8 Safety Data Collection, Recording and Reporting

Safety assessments will consist of monitoring and recording all adverse events and serious adverse events and the regular monitoring of intraoperative vital data by the attending anaesthetist. AE and SAE reporting forms will be provided to each principle investigator by CTC-A. All investigators will be trained on the AE/ SAE definition, documentation and reporting.

8.1 Definition of Adverse Events (AEs)

An adverse event (AE) is defined in the International Conference on Harmonisation (ICH) Guideline for Good Clinical Practice (GCP) as "any untoward medical occurrence, including an exacerbation of a pre-existing condition, in a patient or clinical investigation subject administered a pharmaceutical product and that does not necessarily have a causal relationship with this treatment." (ICH E6:1.2) All AEs will be followed until the event resolves or stabilises at a level acceptable to the investigator.

8.1.1 Instructions for completing adverse event case report forms

Each AE will be reported on an Adverse Event Case Report Form and specified in regard to:

1. Its duration (start and end dates),
2. Its severity grade (mild, moderate, severe)
3. Its relationship to the study drug (suspected / not suspected)
4. Treatment required and action taken with trial drug
5. Outcome
6. Seriousness

Examples of the severity grade, relationship to study treatment and actions taken, as presented in the case report form, are provided below.
The severity grade of an adverse event provides a qualitative assessment of the extent or intensity of an adverse event, as determined by the investigator or as reported by the subject. The severity grade does not reflect the clinical seriousness of the event, only the degree or extent of the affliction or occurrence (e.g. severe nausea, mild seizure), and does not reflect the relationship to study drug.

Severity grade for an adverse event

The intensity of the AE should be judged based on the following

1 = Mild	Awareness of sign(s) or symptom(s) which is/are easily tolerated
2 = Moderate	Enough discomfort to cause interference with usual activity
3 = Severe	Incapacitating or causing inability to work or to perform usual activities

Causal relationship of adverse event

Medical judgment should be used to determine the relationship, considering all relevant factors, including pattern of reaction, temporal relationship, de-challenge or re-challenge, confounding factors such as concomitant medication, concomitant diseases and relevant history. Assessment of causal relationship must be recorded for each adverse event.

Causality will be reported as either “Yes” or “No”.

Yes: There is a reasonable causal relationship between the investigational product administered and the AE.

No: There is no reasonable causal relationship between the investigational product administered and the AE.

8.2 Definition of Serious Adverse Events (SAEs)

A serious adverse event (SAE) is defined as an adverse event that

- Results in death (fatal);
- Is immediately life-threatening;
- Results in persistent or significant disability/incapacity;
- Requires or prolongs patient hospitalisation;
- Is a congenital anomaly/birth defect;

  or
- Based upon appropriate medical judgment, may jeopardise the patient and may require medical or surgical intervention to prevent one of the previously listed outcomes.

A hospitalisation meeting the regulatory definition for “serious” is any inpatient hospital admission that includes a minimum of an overnight stay in a health care facility. Any adverse event that does not meet one of the definitions of serious (eg, emergency room visit, outpatient surgery, or requires urgent investigation) may be considered by the investigator to meet the “other significant medical hazard” criterion for classification as a serious adverse event. Examples include allergic bronchospasm, convulsions, and blood dyscrasias.

An event does not need to be reported as a SAE if it represents only a relapse or an expected change or progression of the condition that was the cause of treatment without any other symptoms and signs than those present before treatment. This type of event needs only to be reported as an AE.

8.3 Reporting Procedures of serious adverse events SAEs

The principle investigator of each centre will have to report all SAEs will be reported by to the sponsor within 24 hours of discovery or notification of the event. The sponsor will collect all SAE reports and write the annual safety report.

9 Study Termination

The study will be prematurely terminated for an individual subject in case of:

- Request of the patient or withdrawal of informed consent
- Patient did not meet the inclusion and/or exclusion criteria
- Patient condition, which is incompatible with a premedication or any study procedure

The study will entirely be terminated in case the risk-benefit-ratio changes in such way, that premature study termination is indicated in order to protect subject’s health.

10 Emergency Procedures

10.1 Emergency Contact Address/Safety Monitor

Prof. Dr. med. Mark Coburn
Managing Senior Physician
Department of Anaesthesia – University Hospital Aachen
Pauwelsstr. 30, D-52074 Aachen, Germany
T +49 241 80 88179
F +49 241 80 82593
E-Mail mcoburn@ukaachen.de

11 Statistics

Primary analysis of the study outcome will be performed according to the intention-to-treat (ITT) principle. The ITT analysis will also include the patients, who have received additional rescue midazolam i.v. preoperatively, on behalf of the attending anaesthetist during the clinical routine.

If prior to the database lock, it appears necessary to define a secondary per protocol (PP) data set, which would be a subset of the ITT data set, composed of all randomised patients who have no major protocol deviations throughout their whole study period, this PP data set would be used for the secondary supportive analysis of the primary efficacy criterion and check of its robustness.

Descriptive analysis of all study data will be performed for both treatment arms. Frequencies for categorical variables and means, medians, standard deviations and selected quantiles for quantitative variables will be tabulated. Distributions of variables will be graphically examined. A subgroup analysis with regard to the gender effects will be performed in addition.

The primary outcome will be analysed with the use of analysis of variance (ANOVA) including treatment effect and study centre as factors. The treatment effect will be tested using an F-test.

The sample size was calculated based on detecting a minimum of 5 unit difference in the primary outcome variable overall patient satisfaction measured with the EVAN-G. Assumptions regarding the standard deviation of EVAN-G in the population was based on

previous work [1]. Setting a type 1 error of 0.05, a power of 0.8 and assuming the standard deviation of EVAN-G to be 14 units, 248 patients per group are needed to detect a 5 unit difference.

Considering a drop-out of 10% and a screening failure of 10%, we decided to include 614 patients in total.

12 Ethical and Legal Aspects

12.1 Independent Ethics Committees

The study will be performed in accordance with the ethical principles that have their origin in the Declaration of Helsinki.

In accordance with the Declaration of Helsinki, the German Medicines Act (Deutsches Arzneimittelgesetz (AMG)) as well as the Good clinical practice (GCP)-guideline the study will be presented to the competent Ethics Committee for RWTH Aachen University and its endorsement will be obtained prior to inclusion of any subject into the study.

In accordance with local legal requirements, study documents also have to be submitted to the respective regulatory authority(ies) for separate approval.

Any change in the study protocol and/or informed consent form will be presented to the named Ethics Committee. They have to be approved by the Ethics Committee before implementation (except for changes in logistics and administration or when necessary to eliminate immediate hazards).

12.2 Authorisation of the Competent Authority

The sponsor will request approval from the respective competent authority (Federal Institute for Drugs and Medical Devices (BfArM), Germany). The trial will only start after the competent authority has approved it. The sponsor will provide copies of the approval documentation to the principle investigator for his files. Any change in the study protocol will be presented to the named Competent Authority. They have to be approved by the Competent Authority before implementation (except for changes in logistics and administration or when necessary to eliminate immediate hazards).

12.3 Notification Requirements

The notification of the clinical trial according to § 67 German Medical Act to the local supervising authority before trial start and in case of any amendments as well as after the

end of the study will with consent of investigator be performed by the sponsor according to SOP. The sponsor will provide a copy of the notification to the principle investigator for his files.

12.4 Informed Consent

According to AMG and GCP-guideline, informed consent will be obtained from patients prior study-participation. The patients will voluntarily confirm their willingness to participate in the study, after comprehensive written and verbally information by an investigator. Patients will be informed about the requirements, concerning data protection and have to agree to the direct access to their individual data. Patients will get ample time and the opportunity to ask questions about the study, before signature. The patients will sign an informed consent form for study participation as well as disclosure of individual data. The informed consent form has to be signed and personally dated by the patient and one of the sub-investigators. The patients will receive a copy of the consent from.

The patient will be informed by a physician in a timely manner if new information becomes available that may be relevant to the patient's willingness to continue participation in the trial. The communication of this information will be documented. The patient will receive a copy of any amendments to the written information and a copy of the signed and dated consent form updates.

Patients will be informed that they are free to withdraw from the study at any time at their own discretion without necessarily giving reasons.

The participation in the clinical trial must be documented on the patient’s health records.

Post-study treatment

No specific post-study arrangements are made and no specific post-study care will be performed after this study. All subjects will return to their standard medical care after the study, as needed. This also applies to subjects who withdraw their consent during the course of the study.

Subject privacy

Patients will be informed about data protection and that data will be pseudonymised and handed to third party anonymised. Access to encoded data or source documents will only be given to authorised bodies or persons (sponsor, authorised staff, auditors, competent authorities or ethics commission) for validation of data. Also in case of publication confidentiality of collected data will be warranted.

Contact point

All subjects will be provided in the informed consent form with a contact address where they may obtain further information regarding clinical studies.

12.5 Duties of the Investigator

The responsibilities of investigator are stated in a separate agreement, which will be prepared by the sponsor; according SOP CTC01 and will be signed by all parties.

12.6 Delegation of Investigator's Duties

The investigator will ensure, that all sub-investigators and the assisting study personnel will be adequately qualified and informed about the study protocol, any amendments, the study medication and their study related responsibilities and functions. The investigator will maintain a study staff authorisation log, where the responsibilities of each person are listed.

12.7 Protocol Changes

The authorisation of relevant competent authority and approval of the ethics committee for any amendments, which will become necessary during the study, will be applied by the sponsor, according to the Standard operating procedures (SOPs).

Reportable amendments are changes, which may affect following aspects:

- Safety of subjects
- Integrity and credibility of data
- Protocol amendment
- Changes in risk evaluation of drugs consisting/ including genetically modified organisms

Every amendment of the protocol has to be signed by the coordinating investigator, the sponsor and the biostatistician.

12.8 Data Protection

All subjects will be identified by a unique randomisation number. Each principle investigator will safely keep a list, which will allow the identification of the pseudonymised patients.

The patient's informed consent, with their printed name and signature will be filed separately in the investigators file.

Monitors, auditors or the competent ethics committee will have access to personal data, but under copies of the subject identification list or an informed consent.

Where required, personal data and health data in particular, may be:

- Hold for inspection by the competent ethics committee for monitoring the orderly performance of the study,
- Passed to other investigators or an authorised party for analysis in a pseudonymised manner.

13 Data Quality Assurance

Inspections by regulatory authority representatives and IECs/IRBs are possible at any time, even after the end of study. The investigator has to inform the sponsor immediately about any inspection. The investigator and institution will permit study-related monitoring, audits, reviews by the IEC/IRB and/or regulatory authorities, and will allow direct access to source data and source documents for such monitoring, audits, and reviews.

13.1 Quality control

Standardisation procedures will be implemented to ensure accurate, consistent, complete, and reliable data, including methods to ensure standardisation among sites (e.g., training, newsletters, investigator meetings, monitoring, centralised evaluations, and validation methods).
To prepare the investigators and to standardise performance, training will be held during an investigators' meeting before study start.
This study will be monitored by a qualified monitor from the CTC-A according to GCP guidelines and the respective SOPs (see Section Monitoring).

13.2 Source documentation requirements

All collected patient data during the course of this clinical study should be entered and/ or filed in the respective patient file (CRF- Case Report Forms). The patient's participation in this study must be appropriately documented in the subject file with study number, subject number, date of subject information, and date of informed consent, date of each visit, and date of the telephone contact. Source data should be filed according to the GCP guidelines.
If the site uses a validated computer system including audit trail with a separate access for the monitor (i.e., the monitor can only access the data of the study subjects), paper printouts are not required. The investigator should not make any changes to these documents.

The sponsor's data management function will be responsible for data processing, in accordance with the sponsor's data management procedures. Database lock will occur only after quality assurance procedures have been completed.

13.3 Data management

All collected data have to be entered in the CRF and to be considered as source data. These include automatic print outs as well as paper-based patient records and electronic patients data.

Investigators will enter the information required by the protocol into an electronic data collection system via internet (eCRF). The eCRF will be developed by the data manager for the study. Detailed information on the eCRF completion will be provided during the site initiation visits. Each site will also be provided with an eCRF completion manual. In general all persons who will enter data into the eCRF will be trained by an e-learning tool. The access to the e-learning tool and to the eCRF is password controlled. Plausibility checks will be performed according to a data validation plan. Inconsistencies in the data will be queried to the investigators via the electronic data collection system; answers to queries or changes of the data will directly be documented in the system. Plausibility checks will be performed to ensure correctness and completeness of these data. The database will be closed, after all data are entered and all queries are solved..

13.4 Direct Access to Source Data

The investigator is obliged to allow study specific monitoring, auditing, and inspections by the competent ethics committee, enable direct access at source data and source documents as well as to support the respective person at his best knowledge.

13.5 Monitoring

This study will be monitored regularly by a qualified monitor from the CTC-A according to GCP guidelines and the respective SOPs. Monitoring procedures include one or more visits designed to clarify all prerequisites before the study commences. Interim monitoring visits will take place on a regular basis according to a mutually agreed schedule.

During these visits, the monitor will check for completion of the entries on the eCRF/CRF; for compliance with the clinical study protocol, ICH-GCP principles, the Declaration of Helsinki, and regulatory authority requirements; for the integrity of the source data with the eCRF/ CRF entries; and for subject eligibility. Monitoring also will be aimed at detecting any misconduct or fraud. In addition, the monitor will check whether all AEs and SAEs have been reported appropriately within the time periods required.

The investigator and all staff will be expected to cooperate with the monitor by providing any missing information whenever possible. The investigator must be available to answer questions arising during regular monitoring visits. In addition, the investigator is required to:

- Have all data properly recorded in the eCRF and subject files prior to each monitoring visit
- Have the source documentation available at the monitoring visits.
- Record all IP dispensed in the eCRF and the drug inventory records.

All subjects who give their informed consent, including those screened, but not entered into the study, will be listed on the subject screening/enrollment log. Further details of monitoring activities will be set forth in the monitoring manual.

13.6 Auditing

Audits will be performed according to the corresponding audit program, including the possibility that a member of the sponsor's quality assurance function may arrange to visit the investigator in order to audit the performance of the study at the study site, as well as all study documents originating there. Auditors conduct their work independently of the clinical study and its performance.

14 Data Handling and Record Keeping

14.1 Conclusion of Documentation

By signing the CRF (eCRF/ eSignature), the investigator confirms that all investigations have been completed and conducted in compliance with the clinical study protocol, and that reliable and complete data have been entered into the eCRF.

14.2 Corrections to data

If corrections are necessary, the subject should be instructed to make a correction by drawing only a single line through the error, leaving the incorrect entry legible. The subject should date the correction and initial it. The investigator should not make any changes to these documents.

The sponsor's data management function will be responsible for data processing, in accordance with the sponsor's data management procedures. Database lock will occur only after quality assurance procedures have been completed.

14.3 Record keeping

Essential documents should be retained at least 10 years

Essential documents at the investigational site include (among other documents):

- Subject files.
- Subject identification code list, which identifies the subject by number, name, and date of birth.
- A signed copy of the final clinical study protocol and any amendment.
- CD/DVD with eCRF data, and any associated subject-related source data (or, where applicable, authorised copies of source data).
- Signed informed consent forms.
- Copies of site investigators' and co-workers' curricula vitae.
- Copies of all direct correspondence with the IEC/IRB and with the regulatory authority(ies).
- Copies of study supply receipt forms and drug inventory forms.
- Copies of all correspondence between the investigator and the monitor, and between the investigator and the sponsor.
- Copies of safety information reported during the study and submitted by the sponsor.

14.4 Archiving of Documents

The investigator will keep the subject's files and original data as long as possible and according to the local methods and facilities. The investigator should maintain the trial documents as specified in the ICH-GCP-Guideline for at least 10 years. The investigator/ institution should take measures to prevent accidental or premature destruction of these documents.

14.5 Destruction of study documents

Study documents may not be destroyed by study site personnel prior to the retention period specified above without the prior written consent of the sponsor. The principal investigator must inform the sponsor in due time if the principal investigator leaves the institution during the retention period. This rule also applies when the institution closes within the retention period.

15 Publication Policy

The study results will be published in appropriate international scientific journals, and publishing details will be given in the clinical study agreement. The study will be registered and study results will be disclosed by the coordinating principal investigator in one or more

public clinical study registry(ies), according to national/international use. The registration will include a list of the investigational sites.

16 Finance and Insurance

16.1 Financing

This clinical trial is an investigator-initiated trial. This trial will be supported by the Department of Anaesthesiology, University Hospital of RWTH Aachen, Germany (costs for regulatory affairs, monitoring and the pharmaceutical products).

16.2 Insurance

A separate patient`s insurance is not completed in accordance with § 40 (1) of the German Medicines Act (AMG).

In the case of fault-based incidences the patients are insured by the general liability insurance of the respective hospital.

A travel accident insurance is not provided and not required.

17 Statement of compliance

Investigational Site(s)

I have thoroughly read and reviewed the clinical study protocol. Having understood the requirements and conditions of the clinical study protocol, I agree to perform the clinical study according to the clinical study protocol, the case report form, ICH-GCP principles (EU Directive 2001/20/EG), the Declaration of Helsinki, and regulatory authority requirements (§40-42 AMG).

I have received the current SmPC (instruction of use). Having been adequately informed about the IP development to date, I also agree to:

- Sign this clinical study protocol before the study formally starts.
- Wait until I have received approval from the appropriate IEC/IRB before enrolling any patient in this study.
- Obtain informed consent for all patients prior to any study-related action performed.
- Start the study only after all legal requirements in my country have been fulfilled.
- Permit study-related monitoring, audits, IEC/IRB review, and regulatory inspections.
- Provide direct access to all study-related records, source documents, and subject files
    - For the monitor, auditor, IEC/IRB, or regulatory authority upon request.
- Use the IP and all study materials only as specified in the clinical study protocol.
- Report to the responsible drug safety officer, within 24 hours, any adverse event (AE) that is serious, whether considered treatment related or not.

Furthermore, I understand that:

- Changes to the clinical study protocol must be made in the form of an amendment that has the prior written approval of RWTH University and – as applicable – of the appropriate IEC/IRB and regulatory authority.
- The content of the clinical study protocol is confidential and proprietary to RWTH University
- Any deviation from the clinical study protocol may lead to early termination of the study site.
- With my signature below, I also acknowledge receipt of the study protocol.

18 Signatures

The study protocol is accepted by

For the Sponsor

**Dr. med. Susanne Isfort**<br>**on behalf of the sponsor´s representative, the coordinating officer of the CTC-A**<br>**University Hospital RWTH Aachen**	Aachen,

The Biostatistician

**András P. Keszei, MD, PhD**<br>**Center for Translational & Clinical Research Aachen (CTC-A)**<br>**RWTH Aachen University Hospital**	Aachen,

Coordinating Investigator

**Prof. Dr. med. Mark Coburn**<br>**Managing Senior Physician**<br>**Department of Anaesthesia**<br>**RWTH Aachen University Hospital**	Aachen,

The Coordinating Investigator`s Representative

**Dr. Ana Kowark (née Stevanovic)**<br>**Consultant**<br>**Department of Anaesthesia**<br>**RWTH Aachen University Hospital**	Aachen,

Principle Investigator

**XX**	Aachen,

19 References

1. Auquier P, Pernoud N, Bruder N, Simeoni M-C, Auffray J-P, Colavolpe C, François G, Gouin F, Manelli J-C, Martin C, Sapin C, Blache J-L: Development and validation of a perioperative satisfaction questionnaire. Anesthesiology 2005; 102:1116-23
2. Kiecolt-Glaser JK, Page GG, Marucha PT, MacCallum RC, Glaser R: Psychological influences on surgical recovery. Perspectives from psychoneuroimmunology. Am Psychol 1998; 53:1209-18
3. Pritchard MJ Identifying and assessing anxiety in pre-operative patients Nursing standard 2009; 23:35-40
4. Machotta A, Schneider G: Prämedikation mit Midazolam: Unerlässlich und gut?(Pro und contra) Der Anaesthesist 2013; 62:225-9
5. Weinbroum AA, Szold O, Ogorek D, Flaishon R: The midazolam-induced paradox phenomenon is reversible by flumazenil. Epidemiology, patient characteristics and review of the literature. Eur J Anaesthesiol 2001; 18:789-97
6. Obiora E, Hubbard R, Sanders RD, Myles PR: The impact of benzodiazepines on occurrence of pneumonia and mortality from pneumonia: a nested case-control and survival analysis in a population-based cohort. Thorax 2013; 68:163-70
7. Sanders RD, Godlee A, Fujimori T, Goulding J, Xin G, Salek-Ardakani S, Snelgrove RJ, Ma D, Maze M, Hussell T: Benzodiazepine Augmented γ-Amino-Butyric Acid Signaling Increases Mortality From Pneumonia in Mice* Critical Care Medicine 2013; 41:1627
8. Inouye SK, Westendorp RG, Saczynski JS: Delirium in elderly people The Lancet 2014; 383:911-22
9. Siddiqi N, Harrison JK, Clegg A, Teale EA, Young J, Taylor J, Simpkins SA: Interventions for preventing delirium in hospitalised non-ICU patients. Cochrane Database Syst Rev 2016; 3:CD005563
10. Sanders RD, Pandharipande PP, Davidson AJ, Ma D, Maze M: Anticipating and managing postoperative delirium and cognitive decline in adults. BMJ 2011; 343:d4331
11. Van Grootven B, Detroyer E, Devriendt E, Sermon A, Deschodt M, Flamaing J, Dubois C, Milisen K: Is preoperative state anxiety a risk factor for postoperative delirium among elderly hip fracture patients? Geriatr Gerontol Int 2016; 16:948-55
12. American Geriatrics Society Expert Panel on Postoperative Delirium in Older Adults American Geriatrics Society abstracted clinical practice guideline for postoperative delirium in older adults. J Am Geriatr Soc 2015; 63:142-50

13. Maurice-Szamburski A, Auquier P, Viarre-Oreal V, Cuvillon P, Carles M, Ripart J, Honore S, Triglia T, Loundou A, Leone M, Bruder N, PremedX Study Investigators: Effect of sedative premedication on patient experience after general anesthesia: a randomized clinical trial. JAMA 2015; 313:916-25
14. Walker KJ, Smith AF: Premedication for anxiety in adult day surgery. Cochrane Database Syst Rev 2009;CD002192
15. Katzman R, Brown T, Fuld P, Peck A, Schechter R, Schimmel H: Validation of a short Orientation-Memory-Concentration Test of cognitive impairment. Am J Psychiatry 1983; 140:734-9
16. Inouye SK, van Dyck CH, Alessi CA, Balkin S, Siegal AP, Horwitz RI: Clarifying confusion: the confusion assessment method. A new method for detection of delirium. Ann Intern Med 1990; 113:941-8
17. Berth H, Petrowski K, Balck F: The Amsterdam Preoperative Anxiety and Information Scale (APAIS) - the first trial of a German version. Psychosoc Med 2007; 4:Doc01
18. EuroQol Group EuroQol--a new facility for the measurement of health-related quality of life. Health Policy 1990; 16:199-208
19. Lawton MP, Brody EM: Assessment of older people: self-maintaining and instrumental activities of daily living. Gerontologist 1969; 9:179-86
20. Oresanya LB, Lyons WL, Finlayson E. Preoperative assessment of the older patient: a narrative review. JAMA 2014; 311: 2110-2
21. K. Alagiakrishnan, T. Marrie, D. Rolfson, W. Coke, R. Camicioli, D. Duggan, B. Launhardt, B. Fisher, D. Gordon, M. Hervas-Malo, and B. Magee, Simple cognitive testing (Mini-Cog) predicts in-hospital delirium in the elderly, J Am Geriatr Soc. 2007; 55: 314-6
22. Podsiadlo D, Richardson S. The timed "Up & Go": a test of basic functional mobility for frail elderly persons. J Am Geriatr Soc 1991; 39: 142-8
23. Mehta RL, Kellum JA, Shah SV, et al.. Acute Kidney Injury Network: report of an initiative to improve outcomes in acute kidney injury. Crit Care. 2007; 11: R31.

20 FIGURE 1

Figure 1 according to the SPIRIT Statement

	STUDY PERIOD					
	**Enrolment**	**Allocation**	**Post-allocation**			**Close-out**
**Visit****	***0***	***1***	***2***	***3***	***4***	***5***
**ENROLMENT:**						
**Eligibility screen**	X					
**Informed consent**	X					
**Allocation**		X				
**INTERVENTIONS:**						
[Midazolam]		X				
[Placebo]		X				
**ASSESSMENTS:**						
**Patients` demographics and medical history *(***age, gender, weight, height, BMI, smoking status, alcohol, ASA)	X					
**Cognitive testing** (SBT)	X				X	X
**Delirium testing (**CAM)	X				X	
**Anxiety** (APAIS)	X					
**Quality of Life** (EQ-5D-5L)	X					X
**Activities of daily living** (IADL)	X					X

**Pain** (VAS)	X		X	X	X	
**Sleeping quality** (VAS)	X				X	
**Well-being** (VAS)	X		X	X	X	
**Frailty** (Mini cog, timed up & go test, history of falls)	X					
**Laboratory values, only if routinely done** (Haematocrit, haemoglobin, creatinine, albumin)	X					
**AEs and SAEs**			X	X	X	
**Patient cooperation** (VAS)			X			
**Anaesthesia related data** (Drugs, type, duration, extubation-time)			X			
**Surgery related data** (Duration, kind and severity)			X			
**Rescue midazolam application**			X			
**Patients vital data on arrival and end of surgery** (SpO_2, RR_{sys}, HR)			X	X (SpO_2)		
**Global patient satisfaction** (EVAN-G)					X	
**Mortality**						X
**Postoperative serious cardiac or pulmonary complications, acute stroke, or acute kidney injury**						X
**Hospital length of stay**						X
**ICU length of stay**						X

AE, adverse event; APAIS, Amsterdam Preoperative Anxiety and Information Scale; ASA, American Society of Anaesthesiologists physical status; BMI, body mass index; CAM, Confusion Assessment

Method; EVAN-G, Evaluation du Vécu de l'Anesthésie Générale, EQ-5D-5L, health-related quality of life assessment; IADL, Instrumental Activities of Daily Living scale; ICU, intensive care unit; RR_{sys}, systolic blood pressure; SAE, serious adverse event; SBT, Short blessed test; SpO_2, peripheral oxygen saturation; VAS, visual analogue scale.

**Visit 0: Preoperative screening and baseline visit, Visit 1: 30-45 minutes before surgery, Visit 2: operating room, Visit 3: surgery day postoperatively within 0.5-1.5 hours after surgery, Visit 4: first postoperative day; Visit 5: 30th postoperative day

18 Signatures

The study protocol is accepted by

For the Sponsor

**Dr. med. Susanne Isfort**<br>**on behalf of the sponsor´s representative, the coordinating officer of the CTC-A**<br>**University Hospital RWTH Aachen**	Aachen, 10.04.2019<br>i.V. [signature]

The Biostatistician

**András P. Keszei, MD, PhD**<br>**Center for Translational & Clinical Research Aachen (CTC-A)**<br>**RWTH Aachen University Hospital**	Aachen, 10.04.2019<br>[signature]

Coordinating Investigator

**Prof. Dr. med. Mark Coburn**<br>**Managing Senior Physician**<br>**Department of Anaesthesia**<br>**RWTH Aachen University Hospital**	Aachen, 23.04.2019<br>[signature]

The Coordinating Investigator`s Representative

**Dr. Ana Kowark (née Stevanovic)**<br>**Consultant**<br>**Department of Anaesthesia**<br>**RWTH Aachen University Hospital**	Aachen, 10.4.19<br>Kowark

Principle Investigator

**XX**	Aachen,
