# Supplementary material for: Preoperative Midazolam and Patient-Centered Outcomes of Older Patients: The I-PROMOTE Randomized Clinical Trial
Source: JAMA Surg. 2023 Dec 20;159(2):129–38. doi: 10.1001/jamasurg.2023.6479 (PMC10733850; doi:10.1001/jamasurg.2023.6479)
Supplement: Supplement 2. — Statistical Analysis Plan [file jamasurg-e236479-s002.pdf]

Supplementary materials to

Preoperative Midazolam and Patient-Centered Outcomes of Older Patients - The I-PROMOTE Randomized Clinical Trial

Ana Kowark, MD; András P Keszei, PhD; Gerhard Schneider, MD; Stefanie Pilge, MD; Frederick Schneider, MD; David P Obert, MD; Marie-Therese Georgii, MD; Markus Heim, MD; Rolf Rossaint, MD; Sebastian Ziemann, MD; Julia van Waesberghe, MD; Michael Czaplik, PhD; Friedrich K Pühringer, MD; Christian Minarski, MD; Verena May, MD; Tobias Malisi, MD; Berthold Drexler, MD; Carmen Maria Ring, MD; Phillip Engler, MD; Roman Tilly, MD; Petra Bischoff, MD; Ulrich Frey, MD; Maria Wittmann, MD; Martin Soehle, MD; Thomas Saller, MD; Peter Kienbaum, MD; Moritz Kretzschmar, PhD; Mark Coburn*, MD; for the I-PROMOTE study group

*Corresponding author. Email: mark.coburn@ukbonn.de
I-PROMOTE-Study group. All contributing collaborators are listed in the Supplement 4.

Supplement 2. Trial Statistical Analysis Plan Version 1, June 25, 2019; based on study protocol Version 2, June 20, 2017.

Statistical Analysis Plan

**Title**	Impact of Midazolam on Perioperative Outcome of Elderly patients: a multicentre randomised controlled trial
**Sponsor**	RWTH Aachen University represented by the Center for Translational & Clinical Research Aachen (CTC-A)
**Sponsor's Representative**	Dr. med. Susanne Isfort<br>Managing Director CTC-A<br>Pauwelsstraße 30, 52074 Aachen, Germany<br>T +49 241 80 80092<br>F +49 241 80 33 80092<br>E-Mail: sisfort@ukaachen.de
**Sponsor`s Study Code**	16-115
**EudraCT- Number**	2016-004555-79
**ClinicalTrials.gov**	NCT03052660
**Investigational Product**	Midazolam
**Principal Coordinating Investigator**	Prof. Dr. med. Mark Coburn, MD<br>Managing Senior Physician<br>Department of Anaesthesia<br>RWTH Aachen University Hospital, Germany
**Biostatistician**	András P. Keszei, MD, PhD CTC-A
**Central Organization/ Project Management**	Dr. med. Ana Stevanovic, MD<br>Consultant, Clinical Research Fellow<br>Department of Anaesthesia<br>RWTH Aachen University Hospital, Germany<br>T +49 241 80 35766 F +49 241 80 33 35766

Based on Protocol Version: V02, 20.06.17

1 Purpose

The following text provides a plan for the analysis of the I-PROMOTE study. The purpose is to describe the methods of analysis and to specify the analysis plan and decisions in advance of the availability of study data.

2 Trial design

Multicentre, randomised, placebo-controlled, double-blinded, two-arm parallel, interventional clinical trial

3 Study objective

3.1 Primary objective

To assess, if preoperative application of placebo compared to midazolam in elderly patients is different with regard to the global postoperative patient satisfaction.

4 Study Plan overview

Figure 1 presents all visits

4.1 Premature study Termination

The study will be prematurely terminated for an individual subject in case of:

- Request of the patient or withdrawal of informed consent
- Patient did not meet the inclusion and/or exclusion criteria
- Patient condition, which is incompatible with a premedication or any study procedure

The study will entirely be terminated in case the risk-benefit-ratio changes in such way, that premature study termination is indicated in order to protect subject's health.

5 Statistical procedures

5.1 Population

Intention-to-treat Population (ITT): This population will be as complete as possible to resemble the ideal ITT population, which consist of all subjects who were randomized in the study. The ITT population will also include the patients, who have received additional "Rescue" i.v. midazolam preoperatively, on behalf of the attending anaesthetist during the clinical routine. According to ICH-E9 guideline patients who received no treatment can be excluded, if the decision to treat or not to treat is not influenced by the knowledge of the

assigned treatment. All reasonable efforts will be made to evaluate the primary endpoint in all study subjects regardless of adherence to the study protocol. Randomized study participants without any post randomization data will be, however, excluded from analysis. The analysis will therefore assume that factors leading to complete lack of post-randomization data are unrelated to the outcome of interest. The exact pre-specification of the full analysis set will be performed based on a blinded data review.

Per-protocol Population (PP): The PP population will consist of a subset of the ITT population composed of patients who have no major protocol deviations throughout their whole study period.

The safety population will include all patients who will have received any study medication.

5.2 Endpoints

5.2.1 Primary endpoint

Global patient satisfaction measured with the EVAN-G questionnaire (1) on the first postoperative day, at visit 4.

5.2.2 Secondary endpoints

- Assessment of preoperative anxiety – assessed by the Visual Analogue Scale
- Assessment of preoperative frailty – performed according to Oresanya et al. (3). This includes in addition to the assessment of the medical history and laboratory values, the history of falls, the Mini-Cog test (4) and the timed "Up & Go" test (5)
- Functional and cognitive recovery – Cognitive status measured by the short blessed test (SBT) (2); Functional ability will be assessed by the Instrumental Activities of Daily Living (IADL) scale (6).
- Postoperative delirium – Confusion Assessment Method (CAM)
- Perioperative condition of well-being, pain and sleeping - assessed by the Visual Analogue Scale
- Patient cooperation directly preoperatively - assessed by the Visual Analogue Scale
- Amount of patients with rescue midazolam application
- Time to extubation
- Health-related quality of life assessment before and 30 days after surgery - measured by the EQ-5D-5L

- Longer-term outcome after 30 days including mortality or the new-onset of serious cardiac or pulmonary complications, acute stroke, or acute kidney injury defined as follows:
  - ***Serious cardiac complication*** <u>Cardiac arrest:</u> The absence of cardiac rhythm or presence of pulseless electrical activity requiring the initiation of CPR, which includes chest compressions. <u>Myocardial infarction:</u> Electrocardiography changes, new elevation in troponin, or physician diagnosis. Signs of myocardial infarction in the autopsy.
  - ***Serious pulmonary complication*** <u>Pneumonia:</u> Clinical or radiological diagnosis. <u>Pulmonary embolism:</u> Radiological diagnosis. Signs of pneumonia or pulmonary embolism in the autopsy
  - ***Acute Stroke*** Defined as a new focal or generalised neurological deficit of >24h duration in motor, sensory, or coordination functions with compatible brain imaging and confirmed by a neurologist. Transient ischemic attack is not considered as acute stroke. Signs of stroke in the autopsy.

    Acute kidney injury Defined according to the AKIN classification as AKI stage ≥2. This means increase of creatinine >2-3x from baseline within the hospital stay. Or urine output less than 0.5 ml kg^{-1} per hour for more than 12 hours. Or signs of acute kidney injury in the autopsy.
- Hospital length of stay (LOS) and intensive care unit (ICU)-LOS

5.2.3 Baseline characteristics

The following presents characteristics that will be described at baseline:

Inclusion Criteria

Legally competent patients; written informed consent prior to study participation; 65-80 years, both genders; elective surgery; expected surgery duration ≥ 30 minutes; planned general or combined regional and general anaesthesia; planned extubation at the end of surgery

Exclusion Criteria

Age > 80 years; age < 65 years; non-fluency in German language; alcohol and/ or drug abuse; chronic benzodiazepine treatment; intracranial surgery; local and stand by anaesthesia or solely regional anaesthesia; monitored anaesthesia care; cardiac surgery; ambulatory surgery; Repeated surgery; contraindications for benzodiazepine application (e.g. sleep apnoea syndrome, severe chronic obstructive pulmonary disease, allergy); allergy against any component of the Placebo (lactose monohydrate, cellulose powder, magnesium stearate, microcrystalline cellulose) or investigational drug (midazolam, lactose) or the capsules (gelatine, E171 titanium dioxide, E132 indigotine); expected benzodiazepine requirement after surgery; expected continuous mandatory ventilation after surgery; patients who explicitly request anxiolytic premedication; patients with severe neurological or psychiatric disorders; Refusal of study participation by the patient; parallel participation in interventional clinical studies within the last 30 days

Demographics

Age, sex, race, height, weight, ASA status

Risk Factors and Relevant Medical History

Pack-years smoking, alcohol consumption, diabetes, arterial hypertension, adipositas, hypercholesterolemia, chronic heart disease, pulmonary disease, renal disease, cerebrovascular disease, malignant disease, previous surgery,

Laboratory measurements

Hematocrit, albumin, creatinine

5.2.4 Safety outcomes

Adverse events

Number of AE, SAE, intensity of AE

Protocol deviation

Number of protocol deviations

5.3 Statistical methods

5.3.1 Demographics and baseline characteristics

Parameters will be described by treatment group using descriptive statistics. Categorical variables will be described using frequencies, and percentages; continuous variables will be summarized using means, medians, standard deviations, minimum, maximum, and 1st and 3rd quartiles. Number of missing observations will also be tabulated.

5.3.2 Primary endpoint analysis

The analysis of the primary endpoint will be the comparison between the two interventions using a linear model including treatment effect, study centre and blocks, but no interaction terms. The treatment effect will be tested using an F-test. The null hypothesis Ho: treatment effect = 0 will be tested against H1: treatment effect ≠ 0 at a 0.05 significance level. 95% confidence intervals for the treatment effect estimate will also be calculated.

5.3.3 Secondary analyses of the primary endpoint

Secondary analyses will be performed in an exploratory manner. A subgroup analysis by gender will be performed using a linear model with treatment effect, study centre, gender and gender by treatment interaction. Treatment effect for both gender will be estimated. A subgroup analysis by frailty status (yes/no) as well as by anxiety status (yes/no) will be performed in a similar manner. Frailty will be defined as described by Oresanya et al. (3) The cut-off value for anxiety will be defined as >12 based on work by Berth et al. (7) (see section 5.3.6.).

In addition, using the method as for the primary analysis, the treatment effect will be estimated in the per-protocol population.

Missing primary endpoint data will be imputed based on age, gender, weight and ASA status using multiple imputation and combined effect estimates will be calculated using Rubin´s formula.

5.3.4 Secondary endpoint analyses

Secondary endpoints will be analyzed in an exploratory manner.

The outcomes functional ability, cognitive recovery, POD, use of rescue midazolam, adverse vital data, presence of long-term outcomes and amnesia will be analyzed as dichotomous outcome variables and the difference in proportions between the treatment groups along with their standard errors will be calculated. For functional ability and cognitive recovery, recovery will be defined as improvement from baseline measurement.

The outcomes well-being, pain, sleeping quality, and patient cooperation, anxiety in the operation room (measured using VAS), health-related quality of life (EQ-5D-5L), length of hospital and ICU stay and time to extubation will be analysed as continuous outcome variables. For well-being, pain, sleeping quality and heatlh-related quality of life change from

baseline will be calculated. The means in each intervention group and differences in means and their standard errors will be calculated.

5.3.5 Adverse events and protocol deviations

Adverse events, and protocol deviations will be listed for each study participant by intervention group. The difference in proportion of patients with AEs and SAEs in the intervention groups will be calculated. The safety population will be used for these analysis.

5.3.6 Computation of outcome parameters

EVAN-G global score

The scores of negatively worded items will be reversed (Items: 8,11,12,13,16,17,18,19,25, and 26) (1). For each individual the mean score for each of the five dimensions (attention, privacy, information, pain, discomfort, and waiting) will be calculated. If fewer than half of the items will be missing, the mean of the nonmissing items will be substituted. The scores will be linearly transformed to a scale from 0 to 100. The global score will be calculated as the mean of the dimension scores.

Short Blessed Test score

The score will be calculated as the weighted sum of the 6 scores defined in the SBT (8). The following weights will be applied: 4, 3, 3, 2, 2, 2.

IADL score

The score will be calculated as the sum of the eight item scores.

APAIS anxiety score

The sum of items 1,2,4, and 5 will be calculated. (7)

EQ-5D-5L score

The EQ-5D-5L index value will be calculated using the German standard value set for the EQ-5D-5L (9,10).

5.4 Other considerations

No interim analyses are planned. The analysis will be carried out using the R language and environment for statistical computing.

Additional analyses may be planned and amended to this plan by the investigators before database lock.

6 Reference

1. Auquier P, Pernoud N, Bruder N, Simeoni M-C, Auffray J-P, Colavolpe C, François G, Gouin F, Manelli J-C, Martin C, Sapin C, Blache J-L: Development and validation of a perioperative satisfaction questionnaire. Anesthesiology 2005; 102:1116-23
2. Morris JC, Heyman A, Mohs RC, Hughes JP, van Belle G, Fillenbaum G, Mellits ED, Clark C. (1989). The Consortium to Establish a Registry for Alzheimer's Disease (CERAD). Part I. Clinical and neuropsychological assessment of Alzheimer's disease. Neurology, 39(9):1159-65.
3. Oresanya LB, Lyons WL, Finlayson E. Preoperative assessment of the older patient: a narrative review. JAMA. 2014;311:2110-20.
4. Alagiakrishnan K, Marrie T, Rolfson D, Coke W, Camicioli R, Duggan D et al. Simple cognitive testing (Mini-Cog) predicts in-hospital delirium in the elderly. J Am Geriatr Soc. 2007;55:314-6.
5. Podsiadlo D, Richardson S. The timed “Up & Go”: a test of basic functional mobility for frail elderly persons. J Am Geriatr Soc. 1991;39:142-8.
6. Lawton MP, Brody EM. Assessment of older people: self-maintaining and instrumental activities of daily living. Gerontologist. 1969;9:179-86.
7. Berth H, Petrowski K, Balck F. The Amsterdam Preoperative Anxiety and Information Scale (APAIS) - the first trial of a German version. Psychosoc Med. 2007;4:Doc01.
8. Katzman R, Brown T, Fuld P, Peck A, Schechter R, Schimmel H. Validation of a short Orientation-Memory-Concentration Test of cognitive impairment. The American journal of psychiatry. 1983;140:734-739.
9. The EuroQol Group (1990). EuroQol-a new facility for the measurement of health-related quality of life. Health Policy 16(3):199-208.
10. Ludwig K, Graf von der Schulenburg JM, Greiner W. German Value Set for the EQ-5D-5L. Pharmacoeconomics. 2018 Feb 19. doi: 10.1007/s40273-018-0615-8.

Figure 1.

	STUDY PERIOD

	Enrolment	Allocation	Post-allocation			Close-out
**Visit****	***0***	***1***	***2***	***3***	***4***	***5***
**ENROLMENT:**						
**Eligibility screen**	X					
**Informed consent**	X					
**Allocation**		X				
**INTERVENTIONS:**						
[Midazolam]		X				
[Placebo]		X				
**ASSESSMENTS:**						
**Patients` demographics and medical history** *(*age, gender, weight, height, BMI, smoking status, alcohol, ASA)	X					
**Cognitive testing** (SBT)	X				X	X
**Delirium testing** (CAM)	X				X	
**Anxiety** (APAIS)	X					
**Quality of Life** (EQ-5D-5L)	X					X
**Activities of daily living** (IADL)	X					X
**Pain** (VAS)	X		X	X	X	
**Sleeping quality** (VAS)	X				X	
**Well-being** (VAS)	X		X	X	X	
**Frailty** (Mini cog, timed up & go test, history of falls)	X					

**Laboratory values, only if routinely done** (Haematocrit, haemoglobin, creatinine, albumin)	X					
**AEs and SAEs**			X	X	X	
**Patient cooperation** (VAS)			X			
**Anaesthesia related data** (Drugs, type, duration, extubation-time)			X			
**Surgery related data** (Duration, kind and severity)			X			
**Rescue midazolam application**			X			
**Patients vital data on arrival and end of surgery** (SpO_2, RR_{sys}, HR)			X	X (SpO_2)		
**Global patient satisfaction** (EVAN-G)					X	
**Mortality**						X
**Postoperative serious cardiac or pulmonary complications, acute stroke, or acute kidney injury**						X
**Hospital length of stay**						X
**ICU length of stay**						X

AE, adverse event; APAIS, Amsterdam Preoperative Anxiety and Information Scale; ASA, American Society of Anaesthesiologists physical status; BMI, body mass index; CAM, Confusion Assessment Method; EVAN-G, Evaluation du Vécu de l'Anesthésie Générale, EQ-5D-5L, health-related quality of life assessment; IADL, Instrumental Activities of Daily Living scale; ICU, intensive care unit; RR_{sys}, systolic blood pressure; SAE, serious adverse event; SBT, Short blessed test; SpO_2, peripheral oxygen saturation; VAS, visual analogue scale.

**Visit 0: Preoperative screening and baseline visit, Visit 1: 30-45 minutes before surgery, Visit 2: operating room, Visit 3: surgery day postoperatively within 0.5-1.5 hours after surgery, Visit 4: first postoperative day; Visit 5: 30th postoperative day
