# Supplementary material for: Preoperative Midazolam and Patient-Centered Outcomes of Older Patients: The I-PROMOTE Randomized Clinical Trial
Source: JAMA Surg. 2023 Dec 20;159(2):129–38. doi: 10.1001/jamasurg.2023.6479 (PMC10733850; doi:10.1001/jamasurg.2023.6479)
Supplement: Supplement 3. — eTable 1. Further Sensitivity Analyses of Primary End Point eTable 2. Perioperative Change of Health-Related Visual Analog Scale Score eFigure 1. Visual Analog Scale for Well-Being eFigure 2. Visual Analog Scale for Pain eFigure 3. Visual Analog Scale for Sleeping Quality eFigure 4. Visual Analog Scale for EuroQol Health eFigure 5. Proportion of Responses by Level Of Severity for the EQ-5D-5L Mobility Dimension at Baseline and at 30-Day Follow-Up eFigure 6. Proportion of Responses by Level of Severity for the EQ-5D-5L Self-Care Dimension at Baseline and at 30-Day Follow-Up eFigure 7. Proportion of Responses by Level of Severity for the EQ-5D-5L Usual Activity Dimension at Baseline and at 30-Day Follow-Up eFigure 8. Proportion of Responses by Level of Severity for the EQ-5D-5L Pain/Discomfort Dimension at Baseline and at 30-Day Follow-Up eFigure 9. Proportion of Responses by Level of Severity for the EQ-5D-5L Anxiety/Depression Dimension at Baseline and at 30-Day Follow-Up eTable 3. EQ-5D-5L Domains With Level of Problems per Point of Time eTable 4. Breakdown of Other Adverse Events [file jamasurg-e236479-s003.pdf]

Supplemental Online Content

Kowark A, Keszei AP, Schneider G, et al; I-PROMOTE Study Group. Preoperative midazolam and patient-centered outcomes of older patients: the I-PROMOTE randomized clinical trial. *JAMA Surg*. Published online December 20, 2023. doi:10.1001/jamasurg.2023.6479

eTable 1. Further Sensitivity Analyses of Primary End Point
eTable 2. Perioperative Change of Health-Related Visual Analog Scale Score
eFigure 1. Visual Analog Scale for Well-Being
eFigure 2. Visual Analog Scale for Pain
eFigure 3. Visual Analog Scale for Sleeping Quality
eFigure 4. Visual Analog Scale for EuroQol Health
eFigure 5. Proportion of Responses by Level Of Severity for the EQ-5D-5L Mobility Dimension at Baseline and at 30-Day Follow-Up
eFigure 6. Proportion of Responses by Level of Severity for the EQ-5D-5L Self-Care Dimension at Baseline and at 30-Day Follow-Up
eFigure 7. Proportion of Responses by Level of Severity for the EQ-5D-5L Usual Activity Dimension at Baseline and at 30-Day Follow-Up
eFigure 8. Proportion of Responses by Level of Severity for the EQ-5D-5L Pain/Discomfort Dimension at Baseline and at 30-Day Follow-Up
eFigure 9. Proportion of Responses by Level of Severity for the EQ-5D-5L Anxiety/Depression Dimension at Baseline and at 30-Day Follow-Up
eTable 3. EQ-5D-5L Domains With Level of Problems per Point of Time
eTable 4. Breakdown of Other Adverse Events

This supplementary material has been provided by the authors to give readers additional information about their work.

© 2023 Kowark A et al. *JAMA Surgery*.

eTable 1. Further Sensitivity Analyses of Primary End Point

	Midazolam	Placebo	Treatment effect (95%CI)	*P*-value
**EVAN-G global index score[a], Mean (SD), Number of patients**				
effect among anxious patients, anxiety cut-off level ≥12[c]	68.2 (7.9), n=64	67.7 (11.5),n=81	0.7 (-2.9 to 4.3)	.70
effect among patients with frailty (with the timed Up & Go test being the marker of the functional status and assumption of albumin levels ≤ 3.3 g/dL for patients with missing data)[d]	66.9 (10.1), n=28	68.6 (9.7), n=14	-2.2 (-9.1 to 4.7)	.53
effect among patients with frailty (with the EQ-5D-5L result for "Self-Care" being the marker of the functional status) [e]	67.6 (9.9), n=31	67.2 (9.5), n=17	-0.2 (-6.6 to 6.1)	.94

Abbreviation: EQ, EuroQuol; EVAN-G, Evaluation du Vécu de l'Anesthésie Generale

[a] Five patients of the total cohort had missing scores in the global satisfaction score (midazolam = 3, placebo = 2).

[b] Evaluation du Vécu de l'Anesthésie Generale (EVAN-G), global patient satisfaction questionnaire comprising 26 items within six dimensions. The scores of negatively worded items were reversed (Items: 8, 11, 12, 13, 16, 17, 18, 19, 25, and 26).[11] For each individual, the mean score for each of the six dimensions (attention, privacy, information, pain, discomfort, and waiting) was calculated. If fewer than half of the items were missing, the mean of the non-missing items was substituted. The scores were linearly transformed to a scale from 0 to 100. The global index score was calculated as the mean of the dimension score.

[c] Anxiety was measured by the Amsterdam Preoperative Anxiety and Information Scale (APAIS).[20] In this sensitivity analysis the most anxious patients were defined as patients having an APAIS score of ≥12.

[d] Patients were defined as frail for this sensitivity analysis, if at least four of the following six markers were present: Mini-Cog total score ≤ 3 points; albumin level of ≤ 3.3 g/dL; > 1 fall in the last 6 months; hematocrit level of < 35%; pre-operative functional status is according to the Timed Up & Go test >15sec. (patient is timed while the patient rises from an arm chair, walks 3 meters, turns, walks back, and sits down again;[13] ≥ 3 comorbidities present and the assumption that the albumin level for patients with missing data was ≤ 3.3 g/dL.

[e] Patients were defined as frail for this sensitivity analysis, if at least four of the following six markers were present: Mini-Cog[12] total score ≤ 3 points; albumin level of ≤ 3.3 g/dL; > 1 fall in the last 6 months; hematocrit level of < 35%; pre-operative functional status is partially dependent or totally dependent (according to the EQ-5D-5L result for "Self-Care" >1);[19] and ≥ 3 comorbidities present.

© 2023 Kowark A et al. *JAMA Surgery*.

eTable 2. Perioperative Change of Health-Related Visual Analog Scale Score

	Midazolam (n=304)	Placebo (n=303)	Difference in means of treatments (SE)
**Change VAS, mean (95% CI)**			
**Baseline to Extubation**			
Well-being	-0.8 (30.7), n=269	-1.7 (31.1), n=262	0.9 (2.7)
Pain	-9.1 (32.7), n=280	-11.0 (30.6), n=277	1.9 (2.7)
**Baseline to PACU/ICU after surgery**			
Well-being	-5.7 (28.6), n=289	-9.2 (28.9), n=285	3.5 (2.4)
Pain	0.1 (34.1), n=299	0.3 (36.2), n=296	-0.2 (2.9)
**Baseline to 1st POD**			
Well-being	-0.6 (24.5), n=301	-2.8 (22.8), n=299	2.2 (1.9)
Pain	-4.0 (31.7), n=301	-1.2 (32.6), n=301	-2.8 (2.6)
Sleep quality	-17.6 (35.1), n=301	-15.4 (36.1), n=300	-2.2 (2.9)
**Baseline to 30th POD**			
EQ health[a]	4.6 (21.0), n=283	1.7 (21.4), n=276	2.9 (1.8)

Abbreviations: EQ, EuroQuol; PACU, Postanesthesia care unit; POD, Postoperative day; SE, Standard error.

Visual analog scale is a psychometric response scale of 100 mm on a paper ranging from 0 to 100.

[a] The vertical visual analog scale was labeled as follows: 100 mm corresponding to ‘The best health you can imagine’ and 0 corresponding to ‘The worst health you can imagine’.

© 2023 Kowark A et al. *JAMA Surgery*.

eFigure 1. Visual Analog Scale for Well-Being

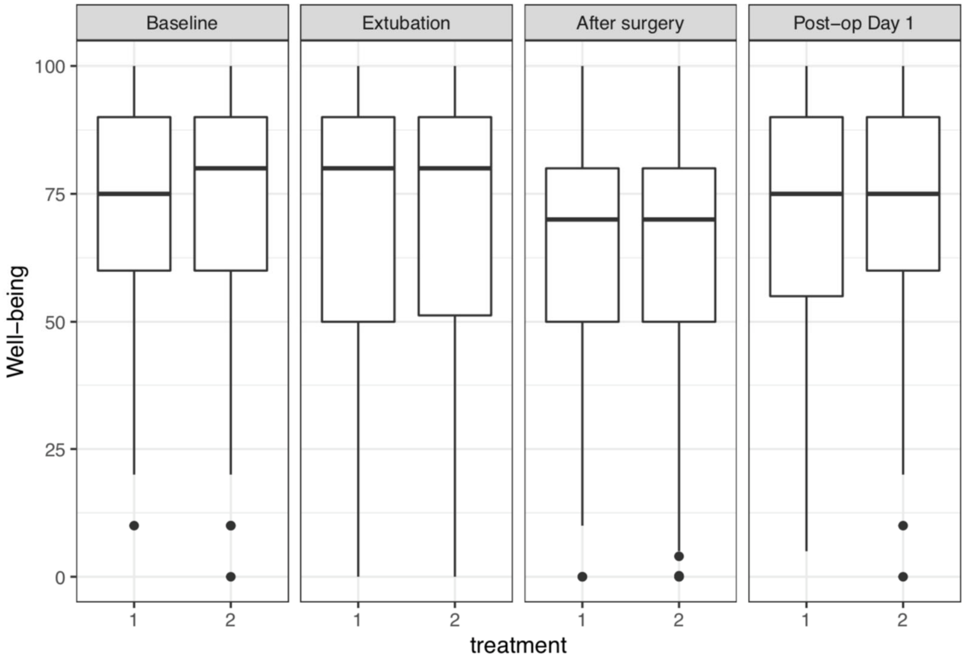

Box-plots for Visual Analog Scale (0-100) values of well-being at different time-points. Treatment 1: midazolam, treatment 2: placebo. The line in the box represents the median, the lower and upper hinges correspond to the first and third quartiles (the 25th and 75th percentiles) and the upper (lower) whiskers extend from the hinge to the largest (smallest) value no further than 1.5 times the interquartile range.

© 2023 Kowark A et al. *JAMA Surgery*.

eFigure 2. Visual Analog Scale for Pain

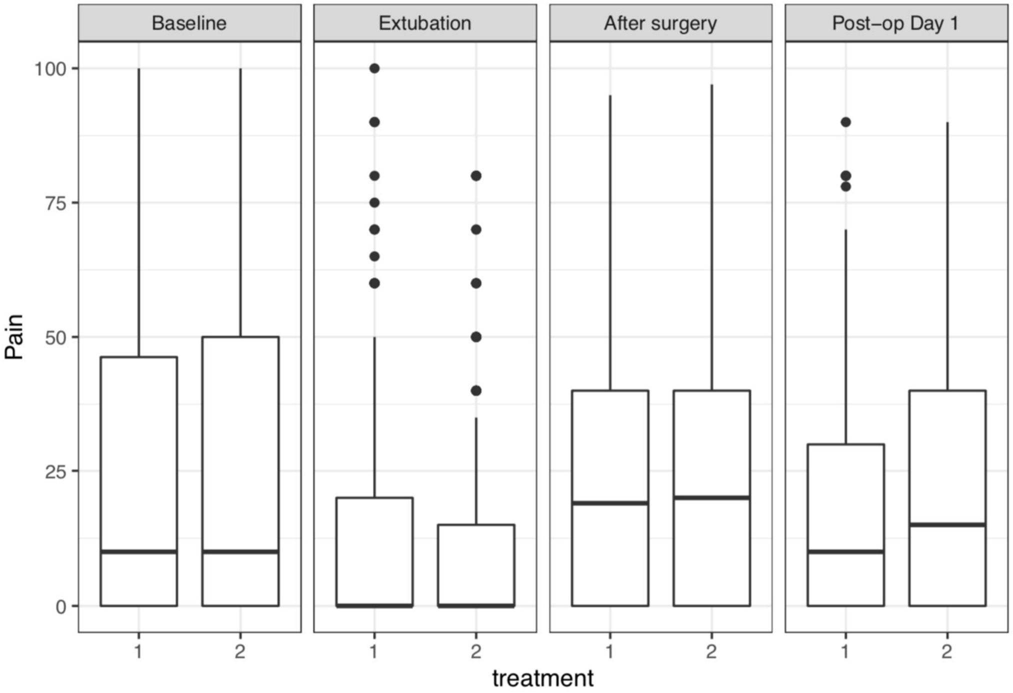

Box-plots for Visual Analog Scale (0-100) values of pain at different time-points. Treatment 1: midazolam, treatment 2: placebo. The line in the box represents the median, the lower and upper hinges correspond to the first and third quartiles (the 25th and 75th percentiles) and the upper (lower) whiskers extend from the hinge to the largest (smallest) value no further than 1.5 times the interquartile range.

© 2023 Kowark A et al. *JAMA Surgery*.

eFigure 3. Visual Analog Scale for Sleeping Quality

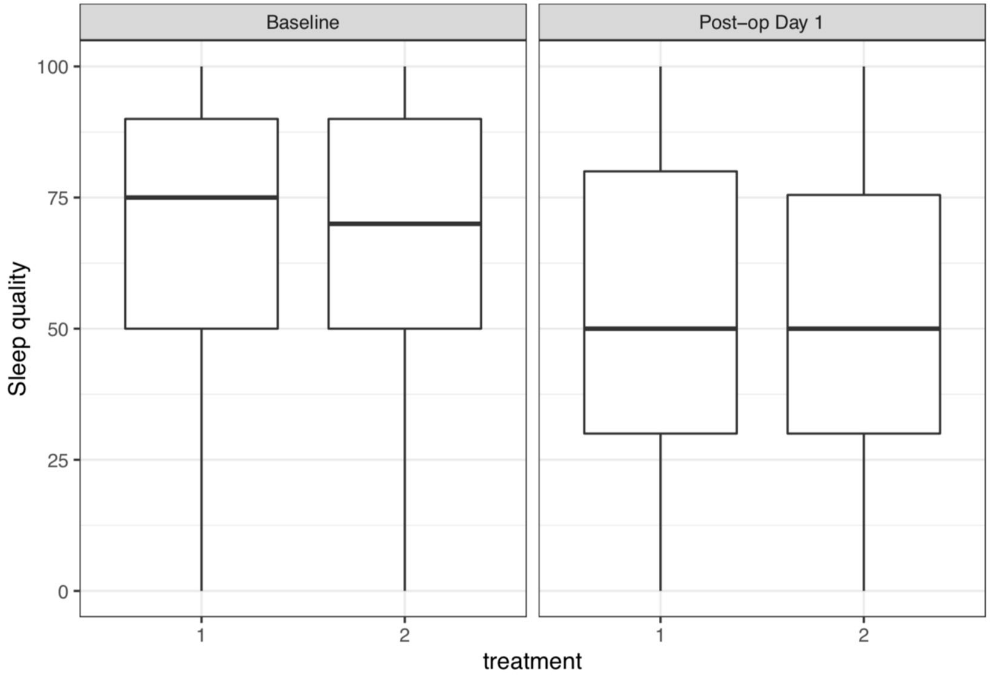

Box-plots for Visual Analog Scale (0-100) values of sleeping-quality at different time-points. Treatment 1: midazolam, treatment 2: placebo. The line in the box represents the median, the lower and upper hinges correspond to the first and third quartiles (the 25th and 75th percentiles) and the upper (lower) whiskers extend from the hinge to the largest (smallest) value no further than 1.5 times the interquartile range.

© 2023 Kowark A et al. *JAMA Surgery*.

eFigure 4. Visual Analog Scale for EuroQol Health

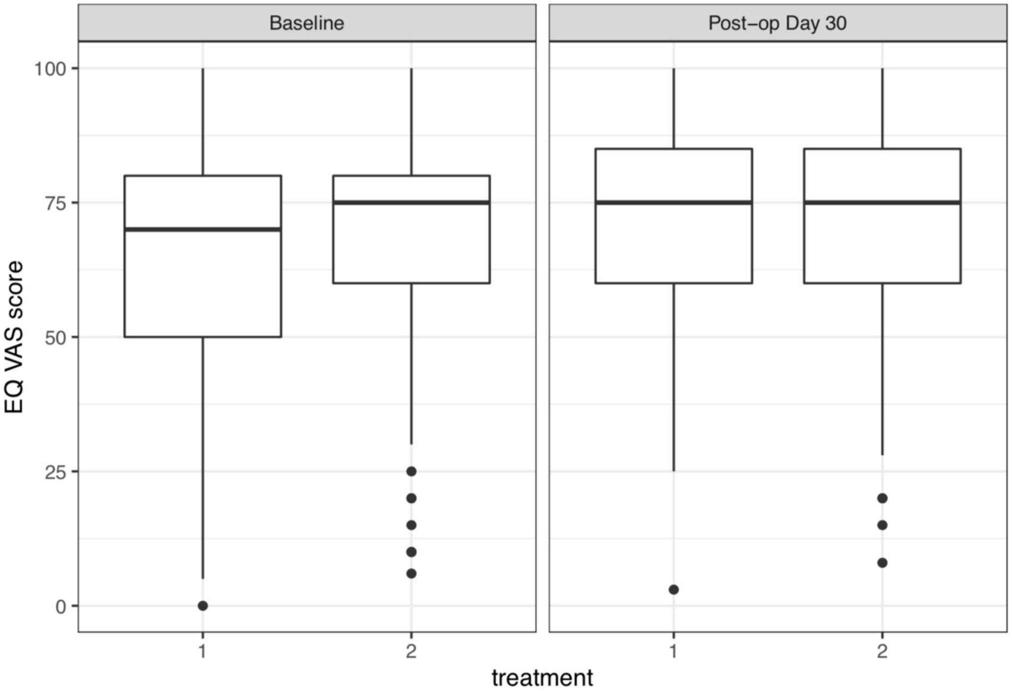

Box-plots for Visual Analog Scale (0-100) values of EuroQol (EQ) Health as part of the EQ-5d-5L[19] quality of life test at two different time-points. Treatment 1: midazolam, treatment 2: placebo. The line in the box represents the median, the lower and upper hinges correspond to the first and third quartiles (the 25th and 75th percentiles) and the upper (lower) whiskers extend from the hinge to the largest (smallest) value no further than 1.5 times the interquartile range.

© 2023 Kowark A et al. *JAMA Surgery*.

eFigure 5. Proportion of Responses by Level Of Severity for the EQ-5D-5L Mobility Dimension at Baseline and at 30-Day Follow-Up

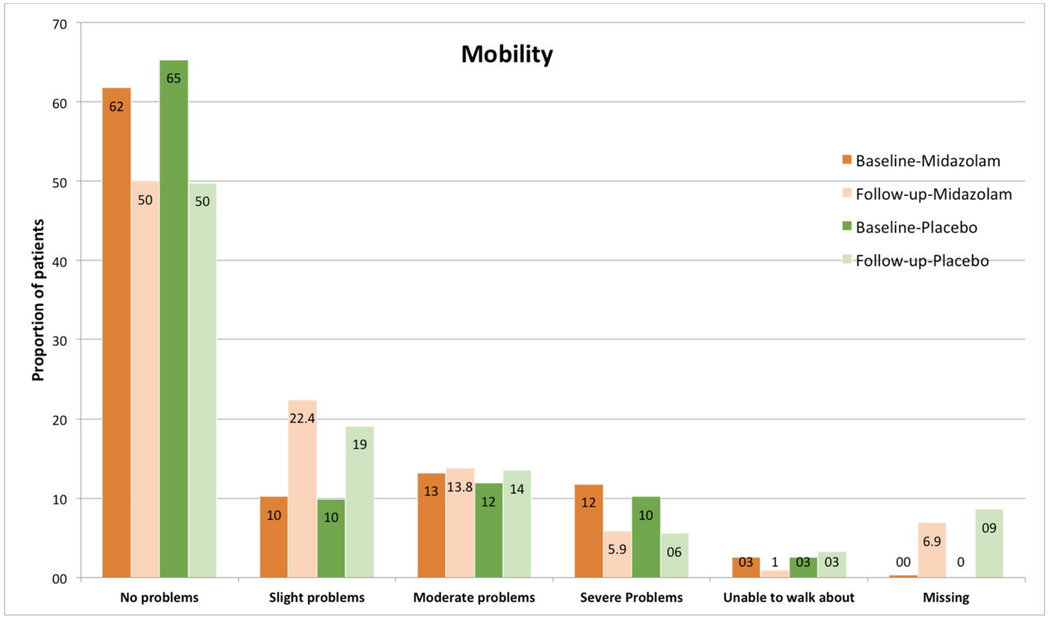

© 2023 Kowark A et al. *JAMA Surgery*.

eFigure 6. Proportion of Responses by Level of Severity for the EQ-5D-5L Self-Care Dimension at Baseline and at 30-Day Follow-Up

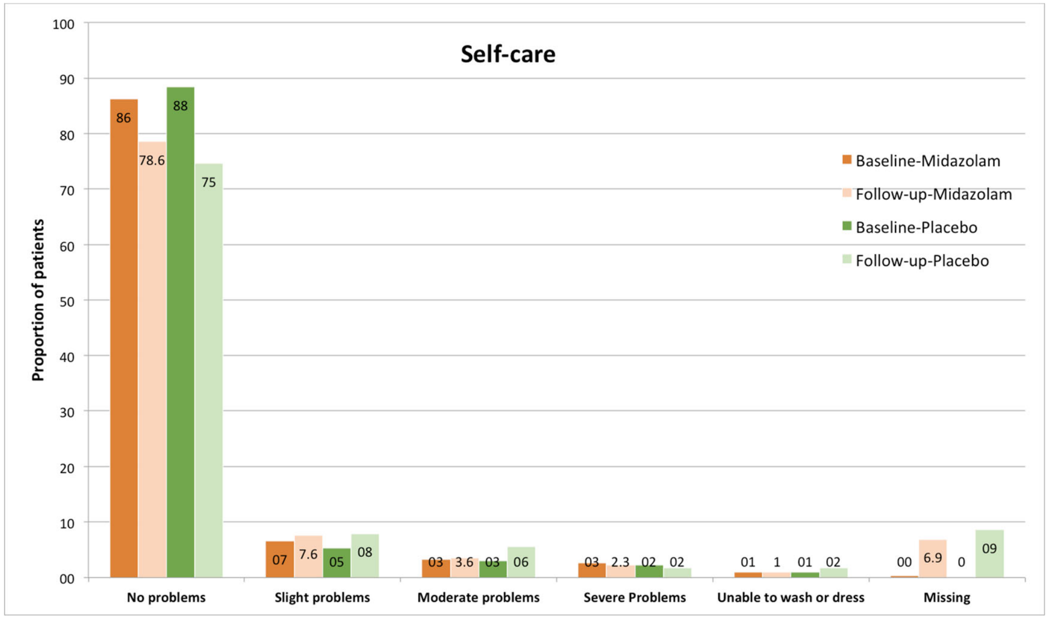

© 2023 Kowark A et al. *JAMA Surgery*.

eFigure 7. Proportion of Responses by Level of Severity for the EQ-5D-5L Usual Activity Dimension at Baseline and at 30-Day Follow-Up

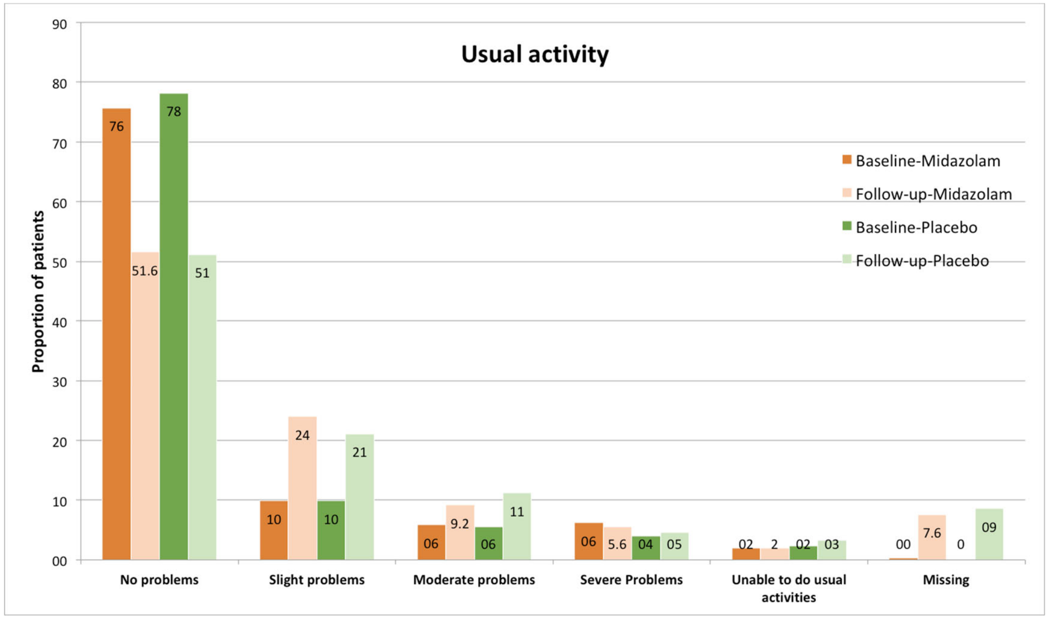

© 2023 Kowark A et al. *JAMA Surgery*.

eFigure 8. Proportion of Responses by Level of Severity for the EQ-5D-5L Pain/Discomfort Dimension at Baseline and at 30-Day Follow-Up

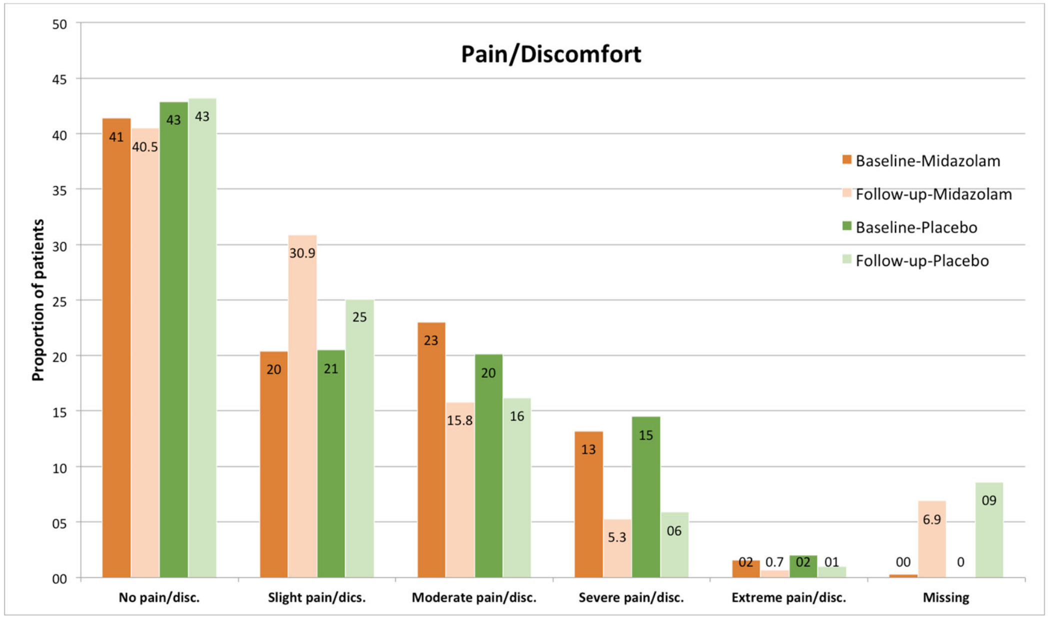

© 2023 Kowark A et al. *JAMA Surgery*.

eFigure 9. Proportion of Responses by Level of Severity for the EQ-5D-5L Anxiety/Depression Dimension at Baseline and at 30-Day Follow-Up

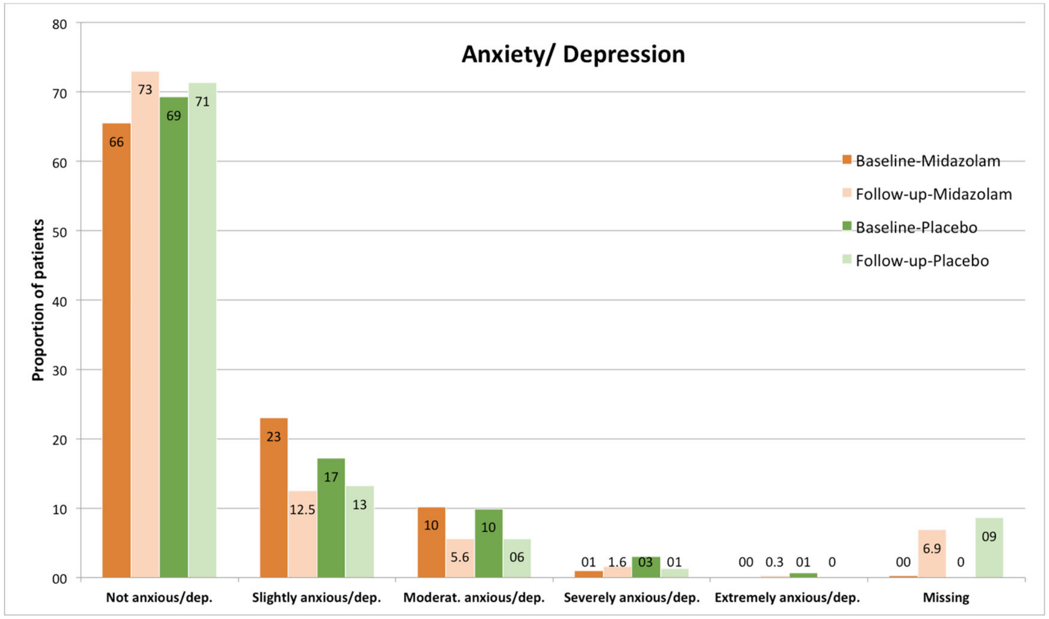

© 2023 Kowark A et al. *JAMA Surgery*.

eTable 3. EQ-5D-5L Domains With Level of Problems per Point of Time

	Midazolam			Placebo		
	**Baseline**	**30-day Follow-up**	***P*-value**	**Baseline**	**30-day Follow-up**	***P*-value**
**Mobility, n (%)**						
No problems	188 (61.8)	152 (50)	.29	198 (65.3)	151 (49.8)	.20
Slight problems	31 (10.2)	68 (22.4)		30 (9.9)	58 (19.1)	
Moderate problems	40 (13.2)	42 (13.8)		36 (11.9)	41 (13.5)	
Severe problems	36 (11.8)	18 (5.9)		31 (10.2)	17 (5.6)	
Unable to walk about	8 (2.6)	3 (1)		8 (2.6)	10 (3.3)	
Missing	1 (0.3)	21 (6.9)		0 (0)	26 (8.6)	
**Self-care, n (%)**						
No problems	262 (86.2)	239 (78.6)	.57	268 (88.4)	226 (74.6)	<.01
Slight problems	20 (6.6)	23 (7.6)		16 (5.3)	24 (7.9)	
Moderate problems	10 (3.3)	11 (3.6)		9 (3.0)	17 (5.6)	
Severe problems	8 (2.6)	7 (2.3)		7 (2.3)	5 (1.7)	
Unable to wash or dress	3 (1)	3 (1)		3 (1.0)	5 (1.7)	
Missing	1 (0.3)	21 (6.9)		0 (0)	26 (8.6)	
**Usual activity, n (%)**						
No problems	230 (75.7)	157 (51.6)	<.001	237 (78.2)	155 (51.2)	
Slight problems	30 (9.9)	73 (24)		30 (9.9)	64 (21.1)	
Moderate problems	18 (5.9)	28 (9.2)		17 (5.6)	34 (11.2)	
Severe problems	19 (6.2)	17 (5.6)		12 (4.0)	14 (4.6)	
Unable to do usual activities	6 (2)	6 (2)		7 (2.3)	10 (3.3)	
Missing	1 (0.3)	23 (7.6)		0 (0)	26 (8.6)	
**Pain/discomfort, n (%)**						
No pain/discomfort	126 (41.4)	123 (40.5)	<.001	130 (42.9)	131 (43.2)	<.01
Slight pain/ discomfort	62 (20.4)	94 (30.9)		62 (20.5)	76 (25.1)	
Moderate pain/ discomfort	70 (23)	48 (15.8)		61 (20.1)	49 (16.2)	
Severe pain/ discomfort	40 (13.2)	16 (5.3)		44 (14.5)	18 (5.9)	
Extreme pain/ discomfort	5 (1.6)	2 (0.7)		6 (2)	3 (1)	

© 2023 Kowark A et al. *JAMA Surgery*.

Missing	1 (0.3)	21 (6.9)		0 (0)	26 (8.6)	
**Anxiety/depression, n (%)**						
Not anxious/depressive	199 (65.5)	222 (73)	<.01	210 (69.3)	216 (71.3)	<.001
Slightly anxious/ depressive	70 (23)	38 (12.5)		52 (17.2)	40 (13.2)	
Moderately anxious/ depressive	31 (10.2)	17 (5.6)		30 (9.9)	17 (5.6)	
Severely anxious/ depressive	3 (1)	5 (1.6)		9 (3)	4 (1.3)	
Extremely anxious/ depressive	0 (0)	1 (0.3)		2 (0.7)	0 (0)	
Missing	1 (0.3)	21 (6.9)		0 (0)	26 (8.6)	

© 2023 Kowark A et al. *JAMA Surgery*.

eTable 4. Breakdown of Other Adverse Events

	**Midazolam (n=304)**	**Placebo (n=303)**
**Single adverse events, n (%)**		
Acute kidney injury	1 (<1)	0
Agitation	0	1 (<1)
Anemia	3 (1)	0
Cardiac arrest	0	1 (<1)
Cardiac failure (acute)	1 (<1)	2 (<1)
Central anticholinergic syndrome	0	1 (<1)
Death	0	1 (<1)
Depression	1 (<1)	0
Dizziness	3 (1)	0
Drowsiness	2 (<1)	0
Extravasation	0	1 (<1)
Eyebrow laceration after falling down in rehabilitation unit	1 (<1)	0
Hypovolemia	0	1 (<1)
Neurologic problem	1 (<1)	0
Pneumonia	2 (<1)	1 (<1)
POCD	0	2 (<1)
PONV	20 (7)	19 (6)
Postoperative bleeding	1 (<1)	2 (<1)
Postoperative delirium	4 (1)	3 (1)
Pulmonary embolism	1 (<1)	0
Re-surgery	0	1 (<1)
Shivering	1 (<1)	0
Stroke	0	2 (<1)
Surgical adverse event	1 (<1)	3 (1)
Syncope	1 (<1)	1 (<1)
Thorax-rigidity	0	1 (<1)

Abbreviation: POCD, postoperative cognitive dysfunction; PONV, postoperative nausea or vomiting

© 2023 Kowark A et al. *JAMA Surgery*.
