# Supplementary material for: Preoperative Midazolam and Patient-Centered Outcomes of Older Patients: The I-PROMOTE Randomized Clinical Trial
Source: JAMA Surg. 2023 Dec 20;159(2):129–38. doi: 10.1001/jamasurg.2023.6479 (PMC10733850; doi:10.1001/jamasurg.2023.6479)
Supplement: Supplement 4. — Group Information. I-PROMOTE Study Group [file jamasurg-e236479-s004.pdf]

Supplemental Online Content: Nonauthor Collaborators

*Indicates required information. Only first name, last name, and suffix will appear in PubMed.

*Group Name(s): I-PROMOTE Study Group							
***First Name and Middle Initial(s)**	***Last Name**	***Suffix (eg, Jr, III)**	Academic Degrees	Institution	Location (city, state/province, country)	Role or Contribution, eg, chair, principal investigator	Group (if more than 1 Group listed in the byline) and/or Subgroup (eg, Steering Committee)
Andres	Brenes		MD	Department of Anesthesiology an	Munich, Germany	sub-investigator	
Leonie	Ernst		MD	Department of Anesthesiology an	Munich, Germany	sub-investigator	
Pia	Feddersen		MD	Department of Anesthesiology an	Munich, Germany	sub-investigator	
Barbara	Kapfer		MD	Department of Anesthesiology an	Munich, Germany	sub-investigator	
Susanne	Maluche		MD	Department of Anesthesiology an	Munich, Germany	sub-investigator	
Ellis	Muggleton		MD	Department of Anesthesiology an	Munich, Germany	sub-investigator	
Michael	Schneider		MD	Department of Anesthesiology an	Munich, Germany	sub-investigator	
Linda	Grüßer		MD	Department of Anesthesiology, M	Aachen, Germany	sub-investigator	
Julia	Wallqvist		MD	Department of Anesthesiology, M	Aachen, Germany	sub-investigator	
Anna	Heusel		MD	Department for Anesthesiology, Intensive Care, Emergency Medicine, Pain Therapy and Palliative Care, Kreiskliniken Reutlingen	Reutlingen, Germany	sub-investigator	
Simon	Diepold		MD	Department for Anesthesiology, Intensive Care, Emergency Medicine, Pain Therapy and Palliative Care, Kreiskliniken Reutlingen	Reutlingen, Germany	sub-investigator	
Christopher	Rex		MD	Department for Anesthesiology, Intensive Care, Emergency Medicine, Pain Therapy and Palliative Care, Kreiskliniken Reutlingen	Reutlingen, Germany	sub-investigator	
Carla	Grundmann		MD	Department of Anesthesiology, Surgical Intensive Care, Pain and Palliative Care, Marien Hospital Herne, University Hospital of Ruhr University Bochum	Herne, Germany	sub-investigator	

*Indicates required information. Only first name, last name, and suffix will appear in PubMed.

***First Name and Middle Initial(s)**	***Last Name**	***Suffix (eg, Jr, III)**	Academic Degrees	Institution	Location (city, state/province, country)	Role or Contribution, eg, chair, principal investigator	Group (if more than 1 Group listed in the byline) and/or Subgroup (eg, Steering Committee)
Jan	Wischermann		MD	Department of Anesthesiology, Surgical Intensive Care, Pain and Palliative Care, Marien Hospital Herne, University Hospital of Ruhr University Bochum	Herne, Germany	sub-investigator	
Louise	Fingerhut		MD	Department of Anesthesiology and Intensive Care Medicine, University Hospital Bonn	Bonn, Germany	sub-investigator	
Claudia	Neumann		MD	Department of Anesthesiology and Intensive Care Medicine, University Hospital Bonn	Bonn, Germany	sub-investigator	
Vera	Guttenthaler		MD	Department of Anesthesiology and Intensive Care Medicine, University Hospital Bonn	Bonn, Germany	sub-investigator	
Josef	Briegel		MD	Department of Anesthesiology, University Hospital, LMU Munich	Munich, Germany	sub-investigator	
Patrick	Möhnle		MD	Department of Anesthesiology, University Hospital, LMU Munich	Munich, Germany	sub-investigator	
Catharina	Lampert		MD	Department of Anesthesiology, University Hospital, LMU Munich	Munich, Germany	sub-investigator	
Tanja	Sulot		MD	Department of Anaesthesiology, University Hospital Düsseldorf	Düsseldorf, Germany	sub-investigator	
