# Supplementary material for: Preoperative Midazolam and Patient-Centered Outcomes of Older Patients: The I-PROMOTE Randomized Clinical Trial
Source: JAMA Surg. 2023 Dec 20;159(2):129–38. doi: 10.1001/jamasurg.2023.6479 (PMC10733850; doi:10.1001/jamasurg.2023.6479)
Supplement: Supplement 5. — Data Sharing Statement [file jamasurg-e236479-s005.pdf]

Data Sharing Statement

Kowark. Preoperative Midazolam and Patient-Centered Outcomes of Older Patients. *JAMA Surg*. Published December 20, 2023. doi:10.1001/jamasurg.2023.6479

Data
Data available: Yes
Data types: Deidentified participant data, Data dictionary
How to access data: Data will be made available upon approval of formal request (methodologically sound proposal including a detailed description of the study objectives, a statistical analysis plan, and a predefined analysis and publication timeline) to the corresponding author. Additional materials might also be required during the process of assessment. The request shall be sent to the corresponding author Prof. Mark Coburn, email address: mark.coburn@ukbonn.de
When available: With publication

Supporting Documents
Document types: Statistical/analytic code
How to access documents: The statistical / analytic code is included in the Trial Statistical Analysis Plan within the Supplementary data of the manuscript.
When available: With publication

Additional Information
Who can access the data: Researchers who provide a methodologically sound proposal to achieve aims in the approved proposal
Types of analyses: Specified purposes only
Mechanisms of data availability: With signed data access agreement.
Any additional restrictions: N/A.
